# Ultratrace Osmium in Wild Mushrooms: Environmental Occurrence and Chemical-Form-Dependent Molecular and Toxicological Interpretation

**DOI:** 10.3390/ijms27188188

**Published:** 2026-09-15

**Authors:** Antonio Peña-Fernández, Hugo Pardo Laurel, Tomás Cámara-Pastor

**Affiliations:** 1 Area of Legal and Forensic Medicine, Department of Surgery, Medical and Social Sciences, Faculty of Medicine and Health Sciences, University of Alcalá, Ctra. Madrid-Barcelona Km, 33.6, 28871 Alcalá de Henares, Madrid, Spain; 2 Leicester School of Allied Health Sciences, De Montfort University, Leicester LE1 9BH, UK; 3 Department of Biomedical Sciences, Faculty of Pharmacy, University of Alcalá, Ctra. Madrid-Barcelona Km, 33.6, 28871 Alcalá de Henares, Madrid, Spain; 4 Scientific Computation Research Institute (SCRIUR), University of La Rioja, 26006 Logroño, La Rioja, Spain

**Keywords:** osmium, wild mushrooms, platinum-group elements, environmental biomonitoring, ICP–MS, ultratrace analysis, analytical background, chemical speciation, osmium tetroxide

## Abstract

Osmium (Os) is an exceptionally scarce platinum-group element whose occurrence in terrestrial biota remains poorly characterised. This study investigated ultratrace Os occurrence in wild mushroom fruiting bodies collected from urban and rural green spaces in Leicester and Leicestershire, UK, as part of a broader environmental biomonitoring programme. An Os-specific analytical audit reconstructed 157 original analytical records into 121 fruiting-body biological units and evaluated occurrence using study-specific procedural-background and low-end calibration criteria. Under the primary analytical interpretation, 28/121 fruiting bodies (23.1%; exact 95% CI: 16.0–31.7%) produced Os-associated responses distinguishable from the operational background criterion, whereas 93/121 were classified as non-detects. Although no fruiting body reached the study-specific operational LLOQ, the occurrence pattern was informative: 58/59 directly comparable classifications were concordant under a stricter same-batch procedural-blank scenario, while broader sensitivity analyses demonstrated that classification at these ultratrace levels remains dependent on analytical-background definition. The findings provide evidence that Os-associated signals occur in wild fungi at extremely low analytical levels and empirically demonstrate how analytical-background definition constrains interpretation at the boundary between occurrence and quantification. Quantitative environmental, exposure and toxicological characterisation will require dedicated Os-specific methods with lower validated quantitative limits, explicit recovery control and preservation of chemical-speciation information.

## 1. Introduction

Osmium (Os; atomic number 76) belongs to the platinum-group elements (PGEs) together with platinum (Pt), palladium (Pd), rhodium (Rh), iridium (Ir) and ruthenium (Ru). The strongly siderophile character of these elements resulted in extensive partitioning into the Earth’s core during planetary differentiation, leaving PGEs among the least abundant elements in the continental crust [1]. Their naturally low environmental concentrations nevertheless coexist with increasingly complex anthropogenic material cycles. Mining and metallurgical processing, specialised chemical and medical applications, fossil-fuel combustion and the use and disposal of PGE-containing technologies can introduce PGEs into surface environmental compartments [1,2]. Environmental archives further demonstrate that anthropogenic activities can substantially alter the natural surficial Os cycle even where absolute Os fluxes remain extremely small [2,3,4].

Osmium is particularly informative from a geochemical perspective because its isotopic composition can distinguish between sources with different geological or anthropogenic signatures. Historical records from peat and sediments have demonstrated progressive anthropogenic overprinting of natural Os inputs, while environmental studies have identified contributions from metallurgical activity, industrial emissions, medical or laboratory waste streams and, under some conditions, automotive sources [2,3,4,5,6]. Consequently, Os occurrence in contemporary terrestrial environments cannot be attributed a priori to a single source. This is particularly relevant in urban systems, where multiple diffuse and point sources may operate simultaneously and where low natural background concentrations make even small anthropogenic contributions potentially detectable [1,6,7].

The environmental chemistry of Os is strongly dependent on oxidation state and chemical form. This point is fundamental because elemental Os, inorganic Os salts, Os oxides and organometallic or coordination compounds differ profoundly in volatility, solubility, reactivity, transport and biological activity [8,9,10]. Osmium tetroxide (OsO_4_), in which Os occurs in the +VIII oxidation state, is a volatile and powerful oxidising agent with well-established irritant and toxic properties toward the eyes, skin and respiratory tract [8,9]. However, environmental determination of total Os after destructive acid digestion does not identify OsO_4_ in the original matrix. Nor does it demonstrate the presence of metallic Os, any particular inorganic species, or any specific coordination complex. Chemical-form-dependent interpretation is therefore essential whenever total environmental Os data are considered in a toxicological context.

Evidence for Os incorporation into terrestrial biological matrices is exceptionally limited. Rodushkin et al. [5] reported Os concentrations and ^187^Os/^188^Os isotope ratios in soils, plants, mushrooms, mosses and lichens from northeast Sweden, describing these as the first Os data for several biological matrices. Their mushroom observations suggested that fungal fruiting bodies may acquire Os from underlying substrates and that its occurrence may vary among fungal taxa and locations. In that study, mushroom groups showed mean Os concentrations in the low pg g^−1^ range, with the highest reported concentration being approximately 30 pg g^−1^ in *Boletus edulis* [5]. A later multielement survey by Mleczek et al. [11] reported Os together with other PGEs in 20 wild mushroom species collected near a heavily trafficked road in Poland, with Os concentrations generally reported at approximately 0.01–0.02 mg kg^−1^ dry weight. Despite substantial methodological differences between these studies, they establish that Os can be detected in fungal fruiting bodies and highlight the paucity of directly comparable data.

More broadly, published information on Os occurrence in fungal matrices remains exceptionally sparse. The limited directly relevant environmental literature has largely originated outside the United Kingdom, including studies of wild mushrooms from Northeast Sweden [5] and Poland [11]. Previous UK biomonitoring of wild edible fungi has included other platinum-group elements, notably Pt, but did not determine Os [12]. To our knowledge, our literature search identified no previous peer-reviewed field study specifically reporting Os occurrence in wild mushroom fruiting bodies collected in England. The present dataset therefore addresses a geographically and analytically under-represented component of terrestrial Os biomonitoring.

Wild mushrooms constitute a useful but analytically and biologically complex biomonitoring matrix. Their elemental composition can reflect substrate geochemistry, element availability, fungal physiology and ecological strategy, the spatial distribution of the underlying mycelium and local environmental heterogeneity. Fruiting-body concentrations therefore cannot be interpreted as a simple function of bulk soil concentration, and opportunistic field collections commonly produce a strongly unbalanced representation of fungal taxa and collection locations. These constraints are especially important for elements such as Os, for which expected environmental concentrations are near the lower analytical capability of many multielement methods.

The analytical determination of environmental Os presents additional challenges. Recent high-sensitivity multielement work in foods has specifically cautioned that ultratrace Os determinations by ICP–MS may be affected by unresolved spectral interferences even when mathematical corrections are applied [13]. At the same time, when environmental responses approach instrumental or procedural background, the choice of procedural-blank population, background correction and operational detection criterion may materially influence classification. Under these circumstances, distinguishing detection from quantitative determination becomes more scientifically informative than assigning numerical concentrations to signals that do not meet defined quantitative criteria.

The toxicological interpretation of Os is equally dependent on chemical identity. In contrast to the sparse environmental literature, an extensive and rapidly expanding biomedical literature concerns deliberately synthesised Os complexes. Osmium (II) arene and related organometallic compounds have been investigated as anticancer agents, with reported mechanisms including mitochondrial dysfunction, reactive oxygen species (ROS) generation, redox perturbation, DNA interactions, protein inhibition, cell-cycle arrest and apoptosis [10,14,15,16,17,18,19,20]. Structure, oxidation state, ligand architecture and intracellular localisation materially alter the biological behaviour of these compounds [10,17,18,19,20]. These mechanistic studies establish that chemically defined Os species can interact with molecular targets, but they cannot be extrapolated quantitatively to digestion-based total Os measured in environmental mushrooms.

A small human biomonitoring literature has also reported total Os in serum in association with reproductive or redox-related outcomes [21,22]. Such observational studies demonstrate that Os can be measured in human biological matrices, but they do not resolve chemical speciation and cannot on their own establish source, exposure route, causal toxicological mechanism or applicability to dietary exposure from mushrooms. Together, the environmental, analytical and biomedical literature therefore points to a critical distinction between elemental occurrence, chemical identity, exposure and molecular effect.

Against this background, the present study forms part of a broader environmental biomonitoring programme designed to characterise metal(loid)s and less-studied technology-relevant elements in wild mushrooms and associated green-space environments from Leicester and Leicestershire, UK. Osmium was included in the original multielement ICP–MS analytical panel with the objective of investigating its environmental occurrence in wild mushroom fruiting bodies. However, subsequent Os-specific auditing of the original analytical records showed that the measured responses lay in an ultratrace domain in which procedural background and low-end calibration performance critically constrained quantitative interpretation. The objectives of the present study were therefore to: (i) reconstruct the Os dataset at the fruiting-body biological-unit level; (ii) determine the occurrence of Os-associated analytical signals using study-specific operational analytical limits; (iii) evaluate the robustness of occurrence classification to alternative procedural- and instrumental-background definitions; (iv) document location- and taxon-specific detection frequencies descriptively, while distinguishing data completeness from patterns suitable for prospective hypothesis testing; and (v) establish the molecular and toxicological conclusions that can, and cannot, be drawn from chemically unresolved elemental Os when no fruiting-body observation meets the operational LLOQ. Accordingly, the primary study endpoint was fruiting-body-level environmental occurrence classification under explicitly defined analytical limits, with sensitivity to analytical-background definition evaluated as part of the interpretation of ultratrace Os responses.

## 2. Results

### 2.1. Dataset Reconstruction and Analytical Performance

The original analytical dataset comprised 157 mushroom records, including measurements from complete fruiting bodies and separately analysed cap and stem portions. Four unmatched tissue-only records could not be reconstructed into complete fruiting-body units and were excluded from the principal biological dataset. The remaining 153 analytical portions comprised 89 whole-fruiting-body records and 64 tissue-specific records forming 32 matched cap–stem pairs. Reconstruction therefore yielded 121 fruiting-body-level biological units, which constituted the environmental unit of analysis. The dataset reconstruction is summarised in Table S1.

Osmium was monitored as ^192^Os within the archived multielement ICP–MS method. External calibration comprised ten non-zero standards from 0.114 to 126.948 µg L^−1^. Linear regression yielded *R*^2^ = 0.999925. Performance at the lowest calibration levels was substantially poorer than in the central and upper calibration range: back-calculation errors were +198.59%, +93.61% and +34.22% at 0.114, 0.228 and 0.570 µg L^−1^, respectively. The 1.141 µg L^−1^ calibrator showed an RSD of 4.00% and a back-calculation error of +15.64% and was therefore retained as the study-specific operational LLOQ. Detailed calibration performance is provided in Table S2.

The primary procedural-background population comprised eight procedure-defined post-replacement acid-digestion blanks: tube IDs 121, 122, 140, 141, 159, 160, 178 and 179. Their median Os response was 0.236003 µg L^−1^. The complete archived blank sequence and the definition of the Scenario A background population are shown in Figure S1 and Table S2. The valid-blank variability used in threshold construction was 0.001002 µg L^−1^, while four instrumental wash measurements yielded an SD of 0.001721 µg L^−1^. The operational detection threshold was 0.005164 µg L^−1^ on the background-adjusted introduced-solution scale and was wash-driven.

Variation in analytical-portion mass produced sample-specific dry-weight analytical limits. Among the 153 included analytical portions, operational detection thresholds ranged from 0.002795 to 0.177502 mg kg^−1^ dw, whereas operational LLOQs ranged from 0.617667 to 39.221875 mg kg^−1^ dw.

The aqueous QCC nominally assigned at 231 µg L^−1^ produced a mean apparent Os of 251.634 µg L^−1^ across two measurements, corresponding to 108.93% of the assigned value, with an RSD of 1.19%. Because this aqueous control was measured above the calibration range applied to the environmental samples, it was interpreted only as a high-level instrumental performance/repeatability check under extrapolated conditions. It does not establish Os-specific matrix recovery, digestion recovery, within-range accuracy or analytical trueness.

The botanical reference material NIST SRM 1570a Trace Elements in Spinach Leaves was available within the wider analytical programme, but its certificate contains no certified reference or information value for Os. Similarly, the other available reference materials did not provide assigned Os concentrations. An Os-specific matrix-recovery or analytical-trueness estimate could therefore not be established.

The principal analytical-performance parameters are summarised in Table 1.

### 2.2. Primary Os Occurrence and Quantitative Non-Estimability

Under the primary Scenario A analytical interpretation, 93/121 fruiting-body units were classified as non-detects, corresponding to 76.9% of the biological dataset (exact 95% CI: 68.3–84.0%). The remaining 28/121 units contained an Os-associated signal above the operational detection threshold but below the study-specific operational LLOQ, corresponding to 23.1% (exact 95% CI: 16.0–31.7%).

No fruiting-body unit yielded a quantitatively valid Os result at or above the operational LLOQ. Consequently, 0/121 units were classified as quantified (0.0%; exact 95% CI: 0.0–3.0%).

**Figure 1:**
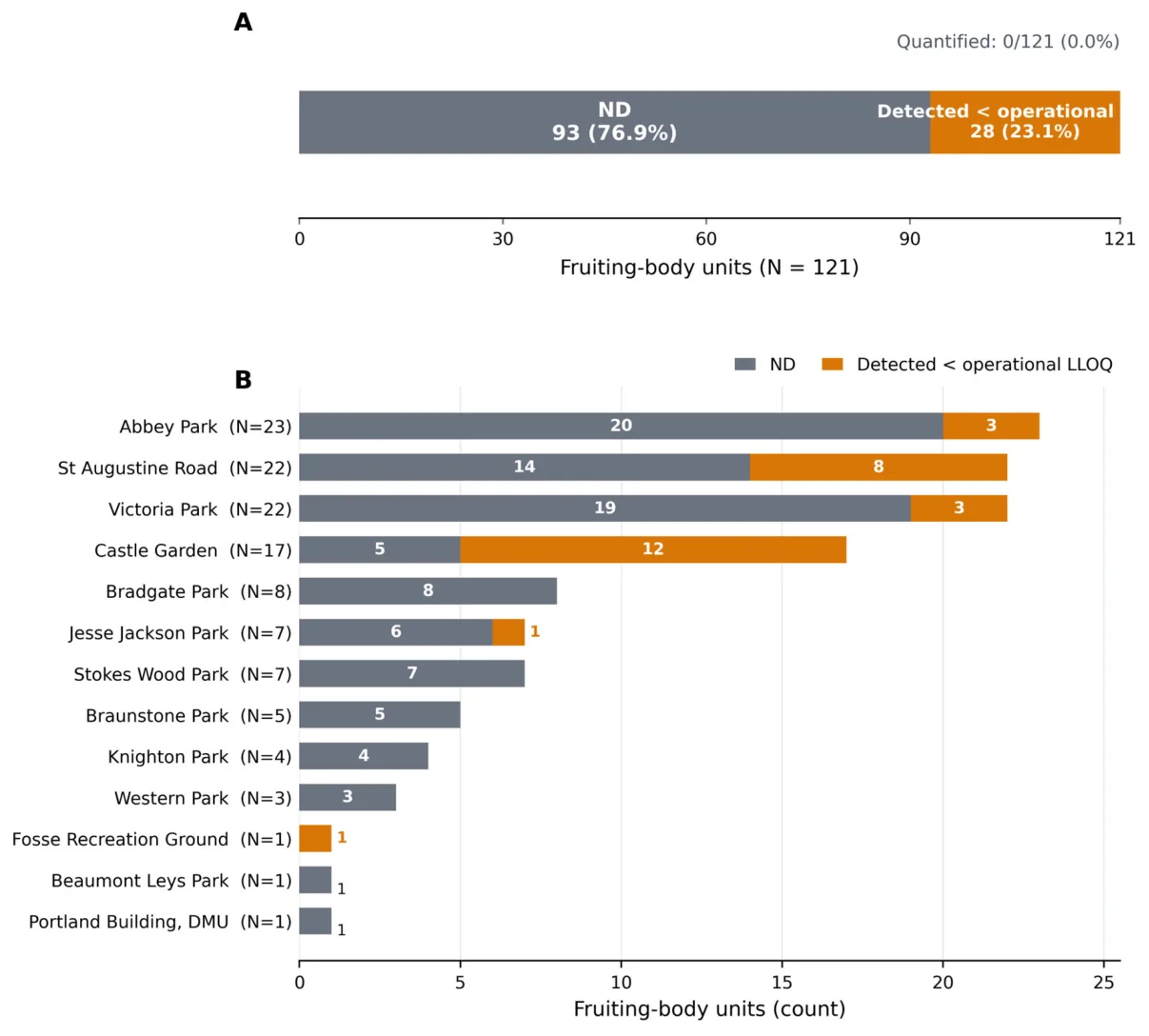
**Os analytical occurrence and classification in wild mushroom fruiting bodies.** (**A**) Overall Scenario A classification among the 121 reconstructed fruiting-body units. (**B**) Location-specific counts of non-detect (ND) and detected-below-operational-LLOQ units, with the location denominator shown in parentheses. Counts rather than percentages are displayed because denominators differed markedly among collection locations. Panel (**B**) is included as a descriptive inventory of the monitored dataset and not as an inferential comparison among locations; collection site, taxonomic composition and analytical batch were unbalanced and partly confounded.

The primary analytical classification is summarised in Table 2 and Figure 1.

Because all 121 fruiting-body observations remained below the study-specific operational LLOQ, there were no uncensored quantitative observations from which a continuous environmental concentration distribution could be estimated defensibly. No mean, median, percentile distribution, regression-on-order-statistics estimate, Kaplan–Meier concentration estimate or parametric concentration model was therefore generated. In particular, no numerical substitution of zero, one-half of an analytical limit, a midpoint or any other surrogate value was assigned to the 28 detected-below-LLOQ fruiting bodies.

This non-estimability was treated as an analytical result rather than as a missing-data problem.

### 2.3. Descriptive Occurrence Profiles for Data Completeness and Hypothesis Generation

Scenario A group-specific detection frequencies were retained to document the composition and analytical-status distribution of the original environmental-monitoring dataset. Because collection location, fungal taxon and analytical batch were strongly unbalanced and partly confounded, these descriptive contrasts were not interpreted as estimates of independent spatial or taxonomic effects. Complete profiles by collection location, final taxonomic assignment, area type, geographical quadrant and analytical batch are provided in Table S3; the main text therefore highlights only those observations that may usefully inform the design of future balanced Os-specific studies.

Castle Garden contained 12 detected-below-operational-LLOQ units among 17 fruiting bodies (70.6%; exact 95% CI: 44.0–89.7%), while St Augustine Road contained 8/22 (36.4%; 17.2–59.3%). Together, these two locations accounted for 20 of the 28 Scenario A detections. However, all 17 Castle Garden fruiting bodies lacked a successful final named-taxon assignment and were represented within a restricted analytical-batch structure. Similarly, *Agaricus bitorquis*, for which 8/22 fruiting bodies were classified as detected below the operational LLOQ, was concentrated at St Augustine Road and within a restricted subset of analytical batches. These frequencies therefore identify observations that can generate testable hypotheses for prospective, balanced sampling rather than evidence of independent location- or taxon-specific Os occurrence.

At the broader area-type level, 28/113 urban fruiting bodies were classified as detected below the operational LLOQ compared with 0/8 rural fruiting bodies. This contrast was retained as a descriptive feature of the monitored dataset but was not interpreted as evidence of urban enrichment because the rural denominator was small and area type was not independent of collection site, taxonomic composition or analytical-batch structure. Complete location-, taxon-, quadrant- and batch-specific profiles, including exact 95% confidence intervals, are provided in Table S3.

The geographical distribution of the 121 fruiting-body classifications is provided in Supplementary Figure S2 as documentation of the sampling context. No independent spatial effect, contamination surface or source attribution is inferred from this descriptive map.

### 2.4. Sensitivity to Analytical-Background Definition

The influence of alternative analytical-background definitions was evaluated using four predefined scenarios.

Scenario A constituted the primary interpretation and used the eight procedure-defined post-replacement acid-digestion blanks. It yielded 93 ND and 28 detected-below-operational-LLOQ fruiting bodies.

Scenario B imposed the stricter requirement for an eligible post-replacement procedural blank from the same analytical batch. This criterion was applicable to 59/121 fruiting-body units. Among these 59 evaluable units, 56 were classified as ND and three as detected below the operational LLOQ; the remaining 62/121 units were not evaluable under Scenario B.

Among the 59 fruiting-body units directly evaluable under both Scenarios A and B, 58/59 classifications were concordant, corresponding to 98.3% agreement (exact 95% CI: 90.9–100.0%). The only discordant unit, FB088, changed from detected below the operational LLOQ under Scenario A to ND under Scenario B. This high concordance applies only to the directly comparable subset and should not be interpreted as independent validation of all Scenario A detections.

Notably, 24 of the 28 Scenario A detections occurred among fruiting-body units for which Scenario B was not evaluable.

Scenario C included all 18 archived acid-digestion blanks, including the earlier procedural phase characterised by elevated background. Under this deliberately conservative sensitivity boundary, all 121/121 fruiting-body units were classified as ND.

Scenario D used wash-derived instrumental background as a diagnostic comparator rather than as an alternative procedural-background definition. Under Scenario D, 103/121 units were classified as ND and 18/121 as detected below the operational LLOQ.

**Figure 2:**
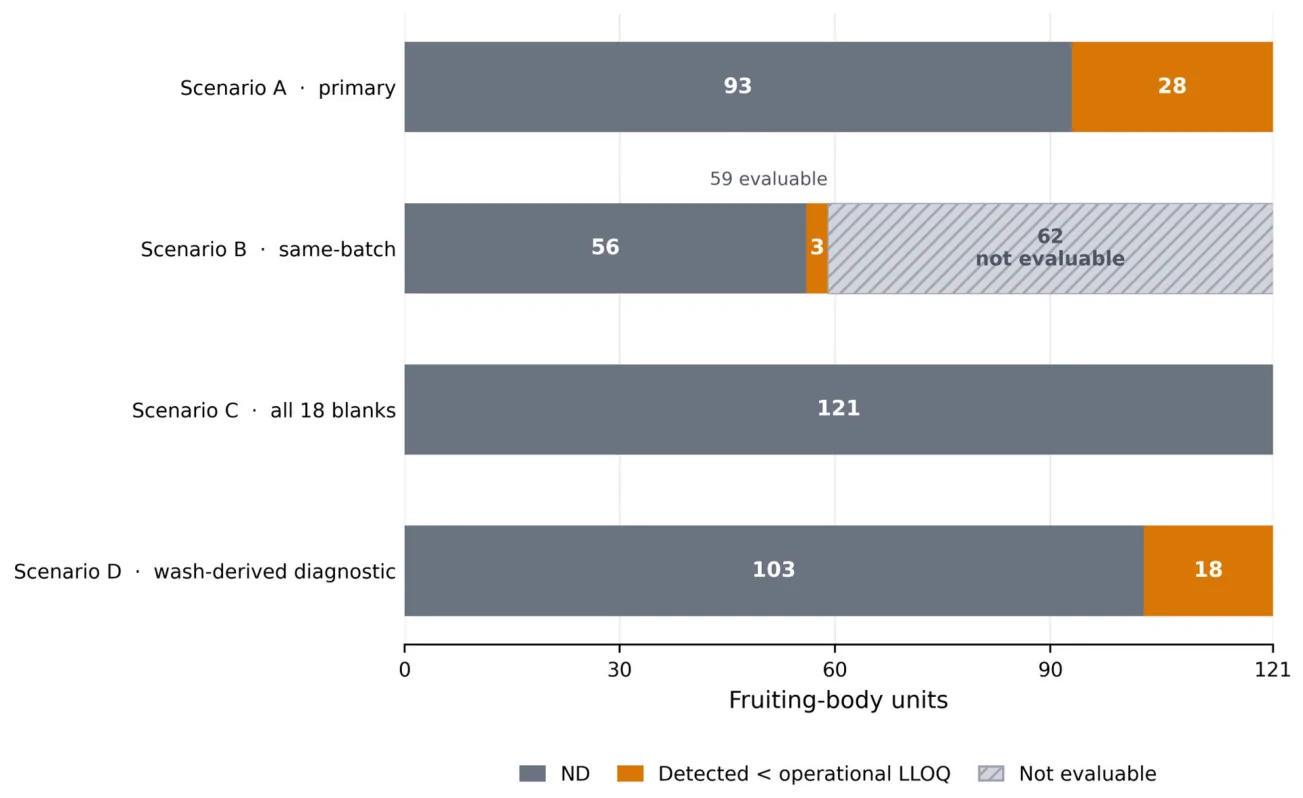
Sensitivity of Os analytical classification to alternative background definitions. Scenario A represents the primary post-replacement procedural-background interpretation; Scenario B requires an eligible post-replacement same-batch procedural blank; Scenario C incorporates all archived acid-digestion blanks and represents a deliberately conservative boundary; Scenario D uses wash-derived instrumental background as a diagnostic comparator. Scenario B contains 59 evaluable and 62 non-evaluable fruiting-body units. Among the 59 fruiting-body units directly comparable under Scenarios A and B, 58/59 classifications were concordant (98.3%; exact 95% CI: 90.9–100.0%); FB088 was the only discordant unit.

The sensitivity results are summarised in Table 3 and Figure 2. Detailed A–B concordance results are provided in Table S4.

Taken together, these analyses demonstrate that classification of Os-associated signals at the observed levels is background-sensitive. The primary Scenario A occurrence result should therefore be interpreted together with, rather than independently from, the analytical-background sensitivity analyses.

### 2.5. Cap–Stem Reconstruction and Contextual Topsoil Programme

Thirty-two fruiting bodies were represented by separately analysed cap and stem portions. Because no Os analytical portion yielded a quantitatively valid result at or above the study-specific operational LLOQ, the dataset did not support quantitative comparison of Os concentrations between cap and stem tissues.

The tissue-specific measurements were therefore used to reconstruct fruiting-body-level analytical classification while preserving the individual fruiting body as the biological unit of analysis. Analytical-portion masses were used in reconstruction and derivation of sample-specific limits; they should not be interpreted as experimentally determined whole-fruiting-body cap:stem biomass proportions.

The wider Leicester/Leicestershire environmental programme also included 850 surface-topsoil increments pooled to generate 26 site-level composite topsoils. Two separately identified physical subsamples were retained from each composite, yielding 52 laboratory records while preserving *N* = 26 as the environmental sample size [23].

Numerically resolved site-level Os concentrations were not available in the final contextual topsoil dataset. In addition, the composite topsoils represented park-scale geochemical conditions rather than substrates collected directly beneath individual fruiting bodies. Consequently, the available data did not support Os soil–mushroom correlations or calculation of soil-to-fungus transfer metrics such as bioconcentration or bioaccumulation factors.

## 3. Discussion

### 3.1. Environmental Occurrence of Os in Wild Mushrooms

The present study identifies Os-associated analytical signals in a subset of wild mushroom fruiting bodies collected from heterogeneous urban and rural green spaces. Under the primary analytical interpretation, 28/121 fruiting-body units produced responses distinguishable from the defined operational background criterion, whereas no unit yielded a quantitatively valid result at or above the study-specific operational LLOQ.

The distinction between these two observations is central. The data support an occurrence classification, but they do not support numerical estimation of the Os concentration distribution in the sampled fungal population.

Published information on Os in mushrooms is extremely sparse. Rodushkin et al. provided the first detailed environmental assessment of Os concentrations and isotopic compositions across multiple biological matrices, including wild mushrooms [5]. Mushroom taxa with relatively less radiogenic Os signatures had a reported mean concentration of approximately 2.5 pg g^−1^, whereas another group had a mean of approximately 11 pg g^−1^. The highest reported mushroom concentration was approximately 30 pg g^−1^ in *Boletus edulis* [5]. Isotopic relationships with the surrounding soils led the authors to propose that mycelial acquisition from soil could contribute substantially to fungal Os, although atmospheric contributions could also differ among taxa.

Mleczek et al. subsequently measured PGEs in 20 wild mushroom species collected near a heavily trafficked road in Poland [11]. Osmium was reported at approximately 0.01–0.02 mg kg^−1^ dw across the analysed species. These concentrations are markedly higher than the pg g^−1^ values reported by Rodushkin et al., illustrating the considerable inter-study differences associated with environmental context, analytical methodology and analytical sensitivity. The principal published mushroom Os observations relevant to interpretation of the present study are summarised in Table 4.

Direct quantitative comparison among these studies remains inappropriate, but the relationship between their analytical domains is informative. Mleczek et al. [11] used microwave-assisted HNO_3_ digestion followed by ICP-OES and reported Os concentrations of approximately 0.01–0.02 mg kg^−1^ dw. These values fall within part of the sample-specific operational detection-threshold range of the present study (0.002795–0.177502 mg kg^−1^ dw) but remain far below its operational LLOQ range (0.617667–39.221875 mg kg^−1^ dw). The partial numerical overlap therefore indicates that Os present at concentrations of the order reported by Mleczek et al. could, for analytical portions with sufficiently low operational detection thresholds, generate a response distinguishable from background in the present dataset. It does not demonstrate quantitative agreement between the two studies because the instrumental platforms, sample-preparation procedures, calibration and background frameworks differed, and no common Os-assigned reference material, interlaboratory comparison or direct cross-method validation was available.

The pg g^−1^ results reported by Rodushkin et al. [5] represent a substantially different analytical domain. Their Os-specific procedure separated Os from the sample matrix through formation of volatile OsO_4_ and coupled gas-phase introduction to double-focusing sector-field ICP–MS, providing analytical sensitivity specifically optimised for ultratrace Os. By comparison, the present environmental-monitoring programme employed quadrupole ICP–MS with ^192^Os acquired in standard mode without collision or reaction gas, using mathematical correction for the ^192^Pt isobaric contribution. The difference between the reported concentration domains should therefore not be interpreted simply as evidence that environmental Os background in northeast Sweden was intrinsically lower. Rodushkin et al. also demonstrated pronounced spatial and isotopic variability consistent with differing natural and anthropogenic Os contributions within the same region [5,6]. Both genuine environmental variability and analytical methodology may therefore contribute to the inter-study differences, but the substantially greater Os-specific sensitivity of the Rodushkin analytical configuration is fundamental to the comparison.

Potential Os sources in Leicester and Leicestershire cannot be assigned from the present data. Automotive catalytic converters are recognised as a potential source of anthropogenic Os, while metallurgical and other industrial activities have produced identifiable Os emissions in other environmental settings [4,6,7]. Previous work from the same Leicester/Leicestershire environmental-monitoring programme has also demonstrated spatially heterogeneous distributions of Cd, Cu and Zn in local green-space topsoils and mushrooms and discussed plausible diffuse urban, traffic-related and historical land-use influences [23]. These observations provide local environmental context for possible anthropogenic inputs but do not constitute Os-specific source evidence. In the absence of Os isotope-ratio measurements, quantitatively resolved spatially paired soil Os data, or other source-specific tracers, attribution of the present mushroom Os-associated signals to road traffic, historical industry or any other individual source would therefore be speculative.

This distinction also prevents a false interpretation of the 93 non-detects. A non-detect under the present operational framework does not establish the absence of Os from the fruiting body. It indicates only that the analytical response was not distinguishable from the applicable operational background criterion. This point is particularly important given published evidence that wild mushrooms can contain Os at concentrations far below the quantitative capability of the current method [5].

### 3.2. Analytical Background, Detection and the Limits of Quantification

The Os dataset illustrates a broader challenge in ultratrace environmental analysis: when analytical responses approach procedural and instrumental background, classification depends not only on the environmental sample but also on how analytical background is defined.

Scenario A used the procedure-defined post-replacement acid-digestion blank population and identified 28 fruiting-body units with above-threshold Os-associated signals. Scenario C, by contrast, incorporated all 18 archived acid-digestion blanks, including the earlier procedural phase characterised by elevated background, and classified all 121 fruiting-body units as ND. The magnitude of this difference demonstrates that the earlier procedural background cannot simply be combined with the later analytical phase without materially altering interpretation of the ultratrace responses.

Scenario B provides a more targeted assessment because it requires an eligible post-replacement procedural blank from the same analytical batch. Among the 59 fruiting-body units for which this comparison was possible, 58 classifications were concordant between Scenarios A and B. This high agreement supports the internal consistency of the primary classification within the directly evaluable subset. However, it does not establish equivalent robustness across the complete dataset, because 24 of the 28 Scenario A detections occurred among fruiting-body units for which Scenario B was not evaluable.

Scenario D provides a complementary instrumental-background diagnostic. Eighteen of the 28 Scenario A detections remained above the wash-derived criterion, whereas ten were reclassified as ND. The most scientifically appropriate interpretation is therefore neither that all 28 Scenario A signals represent unequivocally confirmed environmental Os nor that the detections should be discarded. Rather, the combined evidence supports a background-sensitive ultratrace occurrence pattern whose analytical uncertainty is explicitly demonstrated through alternative background definitions.

The same conservatism applies to quantification. None of the fruiting-body units met the study-specific operational LLOQ. Assigning numerical concentrations to responses solely because they exceeded the operational detection threshold would imply a degree of quantitative confidence not supported by the calibration evidence. Substitution methods would not resolve this limitation. Replacing non-detects or detected-below-LLOQ observations with zero, LLOQ/2, LLOQ/√2 or another fixed surrogate would create numerical information not provided by the experiment and would generate artificial group means, medians and concentration distributions [24]. Quantitative non-estimability was therefore retained as an analytical result rather than treated as a missing-data problem requiring statistical repair.

Determination of Os by multielement ICP–MS introduces additional analytical considerations. Kollander et al. noted that ultratrace Os measurements in food matrices may be affected by unresolved spectral interferences even when mathematical corrections are applied [13]. This reinforces the importance of explicitly documenting the acquisition and interference-correction parameters at m/z 192. In the present study, ^192^Os was acquired in standard mode without collision or reaction gas, and the Syngistix™ acquisition method incorporated mathematical correction for the isobaric contribution of ^192^Pt according to ^192^Os_corr_ = ^192^Os_signal_ − 0.023153 × ^195^Pt. This correction formed part of the original instrumental method and was already incorporated into the exported analytical responses. Nevertheless, correction of a defined isobaric contribution does not remove the broader uncertainties associated with Os determination at ultratrace levels, particularly when sample responses approach instrumental and procedural background.

Osmium volatility represents a further source of analytical uncertainty. In acidic and oxidising media, Os can form volatile species, including OsO_4_, so closed-vessel digestion does not by itself establish quantitative analyte retention. The closed-vessel HNO_3_/H_2_O_2_ digestion used in the present study may have limited physical escape during the 96 °C heating stage relative to an open-vessel procedure, but the magnitude of any volatilisation-related bias during the 12 h digestion cannot be reconstructed from the available data. Potential loss after vessel opening during subsequent filtration, dilution and sample handling also cannot be excluded. Venzago et al. [25] demonstrated that, following oxidative nitric-acid pressure-vessel digestion, an aliquot of the digest could be stabilised specifically for Os using thiourea in combination with ascorbic acid, while the remainder remained available for multielement determination. Their proof-of-concept experiment in Avicel yielded an average Os recovery of 81%. Klose et al. [26] demonstrated complementary stabilisation in biological tissue using ascorbic acid, thiourea and EDTA under non-oxidising preparation conditions, reporting 92% matrix recovery and 99% digestion recovery. These studies demonstrate the feasibility of Os-specific stabilisation strategies, but their recovery values cannot be transferred quantitatively to the present mushroom dataset because the matrices, digestion conditions and analytical procedures differed.

Importantly, no retrospective correction for digestion- or volatilisation-related Os loss was applied. The aqueous Os QCC produced a mean response equivalent to 108.93% of its assigned concentration, with an RSD of 1.19%, but this control did not undergo mushroom digestion or subsequent sample handling and was analysed above the calibration interval used for the environmental samples. It therefore provides evidence only of high-level instrumental response and repeatability and cannot quantify Os recovery or volatilisation loss during sample preparation. None of the available matrix reference materials carried an assigned Os value, and no Os-specific matrix-spike or independent mass-balance experiment was performed. Likewise, the documented ^115^In sequence-level response and Ir (IS1) analyte-specific internal-standard assignment characterise instrumental behaviour but do not measure Os retention through the preceding digestion and post-digestion handling steps. Consequently, the magnitude of any sample-preparation-related Os loss cannot be quantified retrospectively, and no recovery correction was applied.

Because Pd, Pt and Ru were co-acquired with Os in the original multielement sequence, their concurrent analytical responses were also examined as a contextual check on whether the Os observations were compatible with a generally ultratrace PGE environment. The co-measured PGEs likewise occupied the low-response region of their respective analytical domains and therefore provide no evidence of broad PGE enrichment in the mushroom material. This observation is qualitatively consistent with genuinely low Os abundance, but it cannot establish that Os-specific loss during sample preparation was small. Published fungal datasets show that relative PGE abundances vary markedly among species. For example, Mleczek et al. [27] reported 0.021 mg kg^−1^ Os and 5.72 mg kg^−1^ Pt in *Agaricus arvensis*, while Pd and Ru were below detection; *Boletus edulis* contained 0.105 mg kg^−1^ Os and 3.61 mg kg^−1^ Pt, again with Pd and Ru below detection; in contrast, *Laeticutis cristata* contained simultaneously measurable Os, Pt, Pd and Ru at 0.290, 2.90, 0.197 and 0.228 mg kg^−1^, respectively. Among only these examples, the Os/Pt ratio ranges from approximately 0.004 to 0.10, a variation of more than one order of magnitude.

A different multielement survey of commercially cultivated *Lentinula edodes* reported mean concentrations of 0.90 mg kg^−1^ Pt, 0.10 mg kg^−1^ Pd, 0.09 mg kg^−1^ Os and 0.06 mg kg^−1^ Ru [28], corresponding to markedly different Os/Pt, Os/Pd and Os/Ru relationships from those observed in several wild species. These comparisons demonstrate that no conserved inter-PGE ratio can presently be assumed for fungal fruiting bodies. This limitation is consistent with environmental evidence that Os and Pt may become geochemically decoupled because of differences in reactivity and dispersion [29]. Consequently, the low concurrent PGE responses in the present dataset strengthen the qualitative interpretation of an ultratrace PGE setting and provide no indication of a large, generalised PGE burden. They do not, however, provide an independent estimate of the pre-digestion Os concentration, establish that Os-specific recovery was high, or place a quantitative upper bound on volatilisation-related Os loss.

### 3.3. Spatial and Taxonomic Patterns Are Hypothesis-Generating

Descriptively, the primary occurrence classifications were unevenly distributed across collection locations. Castle Garden contributed 12 of the 28 Scenario A detections, and St Augustine Road contributed a further eight. At first sight, the urban/rural profile and northwest-quadrant profile could similarly suggest spatial structuring.

The sampling design does not, however, support causal attribution of these patterns. Castle Garden provides the clearest example. All 17 fruiting bodies from this location lacked a successful final named-taxon assignment, and the site was represented within a restricted analytical-batch structure. Consequently, the apparent Castle Garden signal cannot be separated from taxonomic status or analytical-batch structure.

The same principle applies to *A. bitorquis*, for which 8/22 fruiting bodies were classified as detected below the operational LLOQ. These specimens were concentrated at St Augustine Road and across a restricted subset of analytical batches. The observed frequency cannot therefore establish intrinsic species-specific Os accumulation.

Similarly, the observation that 28/113 urban fruiting bodies were classified as detected below the operational LLOQ, compared with 0/8 rural fruiting bodies, should not be interpreted as evidence of urban enrichment. The rural sample was small, and area type, taxonomic composition, site and analytical batch were not independently balanced. These sources of confounding are documented in Table S5 and Figure S3.

The spatial and taxonomic outputs instead provide hypothesis-generating information for future Os-specific sampling. A dedicated follow-up study could test the apparent patterns using balanced representation of taxa and sites, contemporaneous batch-specific procedural blanks, and substantially lower, independently validated matrix-specific quantitative limits.

### 3.4. Chemical-Form-Dependent Toxicological and Exposure Interpretation

Chemical form is the principal boundary governing toxicological interpretation of the present Os-associated signals. Destructive acid digestion followed by elemental ICP–MS determination does not preserve or identify the original oxidation state, ligand environment, molecular structure or intracellular localisation of Os in mushroom tissue. This distinction is particularly important for osmium tetroxide (OsO_4_), a volatile and strongly oxidising Os(VIII) species associated with pronounced ocular, dermal and respiratory toxicity [8,9]. Nothing in the present elemental measurements demonstrates that OsO_4_ was present in the sampled mushrooms, and its presence cannot be inferred solely from detection of total Os. Environmental and biological matrices may alter Os speciation through reduction, sorption and interaction with inorganic or organic ligands; direct identification of OsO_4_ or any other defined Os species would therefore require species-specific analytical evidence.

The same limitation applies to the extensive biomedical literature on deliberately synthesised Os coordination and organometallic compounds. Defined Os complexes can exhibit substantial and highly structure-dependent biological activity, including altered cellular uptake, redox perturbation, mitochondrial dysfunction, DNA or protein interactions, organelle targeting and regulated cell-death pathways [10,14,15,16,17,18,19,20]. For example, Os(II) arene–azopyridine complexes have been associated with mitochondrial perturbation, cytochrome c release, apoptosis and cell-cycle effects [15], whereas other organo-Os compounds have shown ROS-associated anticancer activity with localisation and biological response dependent on molecular structure [16]. These observations demonstrate that chemically defined Os species can exert molecular effects, but they do not establish the biological activity of chemically unresolved environmental Os. Toxicological evidence derived from OsO_4_ or synthetic Os complexes therefore cannot be transferred quantitatively to the Os-associated signals identified in the present mushroom dataset.

Exposure interpretation is constrained by the same analytical boundary. Because no fruiting-body observation yielded a quantitatively valid Os concentration and the original chemical species were unresolved, neither dietary intake nor a conventional quantitative health-risk metric can be estimated defensibly. The absence of a quantitative risk estimate should not be interpreted as evidence of safety; equally, detection of an Os-associated signal below the operational LLOQ does not by itself constitute evidence of toxicological risk. Potential dietary exposure would additionally depend on consumption patterns, food preparation, chemical stability during processing, gastrointestinal release and bioaccessibility, and subsequent systemic absorption. Human observational studies reporting total serum Os and associations with oxidative-stress, redox or DNA-damage-related markers [21,22] demonstrate that Os can be measured in human biological matrices but do not establish exposure source, route, chemical speciation, causality or a toxicological reference value applicable to mushroom consumption. Progression from environmental occurrence to human-health assessment would therefore require quantitatively validated matrix-specific Os determination, identification of the environmentally relevant Os species, assessment of stability during food preparation, gastrointestinal bioaccessibility and systemic absorption, and a toxicological reference point appropriate to the relevant chemical form and exposure route.

**Figure 3:**
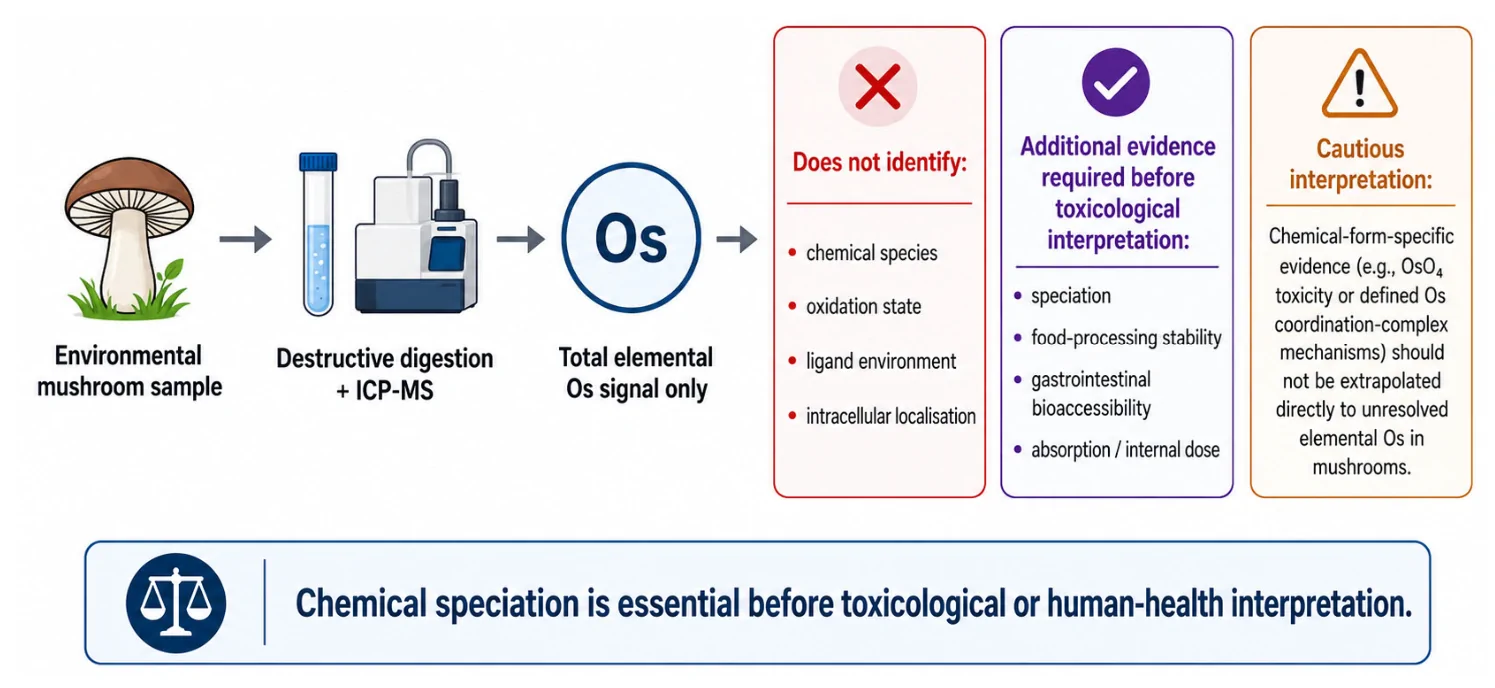
Chemical-form-dependent interpretation of elemental Os occurrence in wild mushrooms. Destructive digestion followed by ICP–MS provides elemental occurrence information but does not preserve chemical speciation. Toxicological or human-health interpretation therefore requires additional evidence on Os chemical form, stability, bioaccessibility, absorption and internal dose.

Figure 3 summarises this interpretive boundary: the present measurements support elemental Os-associated occurrence under the analytical workflow, whereas toxicological or human-health interpretation requires additional chemical-form- and exposure-specific evidence. The toxicological literature is therefore used here to define the limits of inference from environmentally detected Os rather than as evidence that the mushroom observations themselves imply toxicity.

### 3.5. Strengths, Limitations and Research Priorities

The contribution of the present analysis is empirical and environmental rather than the introduction of new analytical principles: it documents Os-associated responses in a subset of prospectively collected wild mushroom samples while quantifying the sensitivity of ultratrace occurrence classification to analytical-background definition. Several limitations define the scope of that evidence. The original multielement method did not provide quantitatively valid Os concentrations at the levels encountered; Os-specific whole-procedure recovery was not established; and, although the original acquisition method incorporated mathematical correction for the isobaric contribution of ^192^Pt, the proximity of the Os responses to procedural and instrumental background remains an intrinsic constraint. In addition, the available topsoils were not spatially paired with individual fruiting bodies, and destructive digestion did not preserve Os chemical speciation. The present dataset therefore supports environmental occurrence classification and analytical-sensitivity assessment, but not a quantitative Os concentration distribution, soil-to-fungus transfer estimates, quantitative exposure or health-risk assessment, or identification of the original Os chemical species.

Future Os-specific fungal biomonitoring should be designed around a predefined matrix-specific target LOQ, rather than a universal minimum sample mass. Under the present workflow, a 0.500 g dry-weight portion, 25 mL final digest volume and ×11 pre-ICP–MS dilution combined with the 1.141 µg L^−1^ operational solution LLOQ correspond to a dry-weight quantitative boundary of approximately 0.63 mg kg^−1^. Achieving a target of 0.01 mg kg^−1^ by increasing sample mass alone, without improving instrumental sensitivity or reducing dilution, would require approximately 31 g dry material and would therefore be impractical. By contrast, Pallavicini et al. demonstrated Os determination in 0.01–0.4 g biological samples using an enriched ^190^Os spike for isotope-dilution analysis coupled to double-focusing sector-field ICP–MS; for a 0.1 g dry sample, method detection limits of approximately 2 pg g^−1^ with solution nebulisation and 0.2 pg g^−1^ with complementary gas-phase introduction were reported [30]. Future mushroom studies should therefore prioritise Os-specific analytical sensitivity and recovery control rather than simply increasing sample mass. A dry mass of approximately 0.1–0.5 g may be operationally sufficient when coupled to an appropriately validated high-sensitivity Os method, but the required mass should ultimately be calculated from the predefined matrix-specific quantitative target.

For analyte tracking and quantification, an enriched Os isotope spike added before digestion, such as ^190^Os for isotope-dilution analysis, would provide a substantially stronger strategy than reliance on a non-isotopic surrogate internal standard alone. Ir may remain useful for monitoring instrumental drift, but it cannot correct Os-specific loss occurring during digestion or post-digestion handling. Future workflows should also incorporate confirmation using more than one Os isotope where analytically feasible, with explicit control of isotope-specific interferences. Matrix validation should include per-batch procedural blanks, Os-specific matrix spikes across the intended concentration range and a homogenised mushroom-matrix quality-control material characterised against an Os-specific reference method; where no certified mushroom material with an assigned Os value is available, an in-house matrix control assigned by isotope-dilution ICP–MS would provide a practical alternative. Stabilisation strategies such as thiourea/ascorbic acid or related validated approaches should be incorporated and evaluated specifically in mushroom digestates [25,26]. Finally, total-Os determination and chemical-speciation analysis should be treated as separate analytical objectives: destructive digestion can support total-Os measurement but cannot preserve the original species, whereas chemical-form-specific work would require species-preserving extraction, separation and detection strategies with explicit assessment of species stability and recovery. No validated mushroom-specific Os speciation protocol is presently established, so such methods would require prospective development and validation rather than retrospective inference from total elemental Os.

## 4. Materials and Methods

### 4.1. Study Area and Mushroom Sampling

Wild mushroom fruiting bodies were collected from public green spaces in Leicester and selected rural locations in Leicestershire, East Midlands, United Kingdom, as part of a broader environmental-biomonitoring programme.

Field sampling was conducted on 12 dates between 12 September and 18 November 2019. Collections were performed under dry conditions following approximately 24 h without rainfall, with recorded ambient temperatures ranging from approximately 8 to 17 °C. Fruiting bodies occurring naturally within accessible areas of the selected green spaces were located visually, and the geographical position of each collection point was recorded using a handheld Global Positioning System (GPS) device.

Only fresh, young and macroscopically intact fruiting bodies were selected. Specimens showing substantial decomposition, insect damage, visible parasitism, trampling or other marked deterioration were excluded, as were fruiting bodies growing in conspicuously littered or heavily disturbed microsites.

Nitrile gloves were worn throughout collection to minimise external contamination and to avoid direct contact with potentially toxic or inedible taxa. Complete fruiting bodies were collected where practicable, individually placed in labelled, sealed polypropylene bags and transported to the laboratory for taxonomic assessment and trace-element sample preparation.

Sampling reflected the natural availability of fruiting bodies and was not designed as a balanced taxonomic experiment. No minimum separation distance was imposed between specimens, and neither the genotypic identity nor the spatial extent of the underlying fungal mycelia was determined. Consequently, multiple fruiting bodies collected from the same location cannot necessarily be assumed to represent independent fungal genets.

### 4.2. Taxonomic Identification and Molecular DNA Barcoding

Taxonomic identification was based initially on macroscopic characteristics of the collected fruiting bodies and was subsequently supported by molecular DNA barcoding using the fungal ribosomal internal transcribed spacer (ITS) region.

Approximately 100 mg of frozen mushroom material was cryogenically homogenised with liquid nitrogen using a ceramic mortar and pestle. Genomic DNA was extracted using the DNeasy Plant Mini Kit^®^ (QIAGEN Inc., Germantown, MD, USA) according to the manufacturer’s instructions and the laboratory workflow previously described by Sgamma et al. [31].

DNA concentration and purity were assessed spectrophotometrically using a NanoDrop Lite Spectrophotometer (Thermo Fisher Scientific, Wilmington, DE, USA). The fungal ITS region was amplified using the universal ITS1 and ITS4 primers [32], consistent with the established use of ITS as the principal universal fungal DNA barcode [33]. Primer sequences were ITS1, 5′-TCCGTAGGTGAACCTGCGG-3′ and ITS4, 5′-TCCTCCGCTTATTGATATGC-3′.

PCR amplification was performed using 1× MyTaq Red Mix (Bioline, London, UK), 0.2 µM of each ITS1 and ITS4 primer and 1 µL of genomic DNA template, following the established laboratory workflow described by Sgamma et al. [31]. Thermocycling was performed using a StepOnePlus™ Real-Time PCR System (Applied Biosystems, Foster City, CA, USA), with an initial denaturation at 94 °C for 2 min, followed by 40 cycles of 94 °C for 15 s, 60 °C for 30 s and 72 °C for 30 s, and a final extension at 72 °C for 2 min. Amplified products were assessed by agarose-gel electrophoresis, purified and submitted to Macrogen Europe B.V. (Amsterdam, The Netherlands) for Sanger sequencing.

Sequence reads were assembled into consensus ITS sequences using CLC Main Workbench version 7.5.1 (QIAGEN, Aarhus, Denmark) and subsequently interrogated against reference nucleotide sequences using the National Centre for Biotechnology Information Basic Local Alignment Search Tool (NCBI BLAST, Bethesda, MD, USA; accessed in 2022) [34].

At the reconstructed fruiting-body level, successful ITS amplification and interpretable sequence information were obtained for 104/121 units (86.0%), corresponding to specimens assigned to named taxa. For the remaining 17/121 units, DNA extraction was performed, but repeated PCR did not yield an amplicon suitable for sequence-based assignment. These specimens were retained as unknown/unidentified taxa in the complete analytical dataset.

No new numerical sequence-identity or query-coverage threshold was imposed retrospectively; the present study retained the original ITS-supported taxonomic assignments.

### 4.3. Cleaning, Tissue Separation and Homogenisation

Visible debris, insects and adherent soil particles were removed manually from the fruiting-body surfaces. Samples were washed with tap water, rinsed with deionised water and finally rinsed with Milli-Q^®^ ultrapure water produced using a Milli-Q^®^ Direct 8 water purification system (Millipore SAS, Molsheim, France).

Where sufficient biomass was available, fruiting bodies were separated into cap and stem fractions before drying to preserve tissue-specific analytical information. Specimens with insufficient material for separation were processed as whole fruiting bodies.

Cleaned material was oven-dried at 50 °C until constant weight, typically overnight, using laboratory drying equipment supplied by Fisher Scientific UK Ltd. (Loughborough, Leicestershire, UK). Dried mushroom material was cryogenically homogenised with liquid nitrogen using a ceramic mortar and pestle until a fine, visually homogeneous powder was obtained. No sieving step was applied.

Homogenised samples were thoroughly mixed, transferred to pre-cleaned capped polypropylene tubes and stored at −80 °C in laboratory freezing equipment supplied by Fisher Scientific UK Ltd. (Loughborough, Leicestershire, UK) until digestion. Reusable sample-contact equipment was cleaned between specimens using laboratory detergent where appropriate, followed by Milli-Q^®^ ultrapure water, 5% HNO_3_ and repeated rinsing with Milli-Q^®^ ultrapure water. The wider sample-preparation workflow has previously been described for the associated Leicester mushroom programme [35].

### 4.4. Acid Digestion and Dry-Weight Reconstruction

Dried, homogenised mushroom material was subjected to closed-vessel, non-microwave acid digestion in pre-cleaned Teflon digestion vessels. Unless otherwise specified, consumables used for digestion and filtration were supplied by Fisher Scientific UK Ltd. (Loughborough, Leicestershire, UK).

Where sufficient biomass was available, approximately 0.500 g dry weight of homogenised mushroom material was accurately weighed into each digestion vessel. Smaller analytical portions were retained where sample availability was limited, and the exact dry mass of each analytical portion was incorporated individually into the subsequent dry-weight analytical-limit calculations.

Five millilitres of 65% HNO_3_ (Suprapur^®^ grade) were added to each analytical portion, followed by 1 mL of 30% H_2_O_2_ (Suprapur^®^ grade); both reagents were supplied by Fisher Scientific UK Ltd. (Loughborough, Leicestershire, UK). The sealed digestion vessels were maintained at room temperature for approximately 8 h to permit pre-digestion and were subsequently heated at 96 °C for 12 h.

After cooling to room temperature, digestates were filtered through Whatman™ Grade 42 ashless quantitative filter paper and quantitatively diluted to a final volume of 25 mL with Milli-Q^®^ ultrapure water. Before instrumental measurement, an aliquot of each digest was additionally passed through a 0.45 µm PTFE syringe filter.

Immediately before ICP–MS analysis, mushroom digestates were manually diluted 11-fold. No additional prepFAST dilution was applied to these records. The ×11 pre-instrument dilution factor was therefore included once, and only once, in the analytical reconstruction.

For a quantitatively valid response, the corresponding dry-weight Os concentration would be calculated as:

*C*_Os_ (ng g^−1^ dw) = [C_introduced_ (µg L^−1^) × 11 × 25]/m

where *C*_introduced_ represents the relevant Os concentration in the solution introduced into the ICP–MS, 11 is the manual pre-instrument dilution factor, 25 is the final digest volume in millilitres, and *m* is the analytical-portion dry mass in grams.

The same conversion framework was applied to the solution-scale operational detection threshold and operational LLOQ, thereby generating analytical-portion-specific dry-weight limits.

For observations below the operational LLOQ, this calculation was used solely to establish the corresponding analytical bounds and was not used to assign reportable Os concentrations.

### 4.5. ICP–MS Determination, Calibration and Quality Assurance

All ICP–MS measurements, external calibration, internal-standard monitoring and associated instrumental quality-assurance procedures were performed at the Geography Research and Teaching Laboratories, School of Geography and the Environment, University of Oxford, Oxford, UK. Mushroom digestates were analysed using a NexION^®^ 2000B ICP–MS system (PerkinElmer, Waltham, MA, USA) operated using Syngistix™ Software for ICP-MS, version 3.2.

Osmium was monitored at *m*/*z* 192 as ^192^Os. The Syngistix™ acquisition method used for the PGE measurements incorporated mathematical correction for the isobaric contribution of ^192^Pt to the ^192^Os response according to ^192^Os_corr_ = ^192^Os_signal_ − 0.023153 × ^195^Pt. This correction formed part of the original instrumental acquisition method and was therefore already incorporated into the Syngistix-generated analytical output; it was not reapplied during the present analytical reconstruction.

The ^192^Os acquisition was performed in peak-hopping standard mode without collision or reaction gas, using a nominal mass setting of 191.962, 20 sweeps per reading, one reading per replicate and five instrumental replicates. The dwell time was 25 ms per AMU, corresponding to an integration time of 500 ms. Each exported analytical record therefore represented the instrumentally averaged result of five replicates.

External calibration performed at the University of Oxford used commercially prepared customised multielement standards supplied by QMx Laboratories, containing the analyte suite required for the analytical programme. These standards were serially diluted to generate a ten-point non-zero calibration for Os at nominal concentrations of 0.114, 0.228, 0.570, 1.141, 2.852, 5.078, 10.156, 25.390, 50.779 and 126.948 µg L^−1^. Calibration solutions and environmental samples were analysed within the same analytical day to minimise effects of instrumental drift and changing laboratory conditions. According to the laboratory information available for the analytical programme, calibration *R*^2^ values exceeded 0.995 for all included elements. For Os, linear regression of net analytical intensity against nominal concentration using a free intercept yielded *R*^2^ = 0.999925. An independently sourced commercial multielement standard supplied by Scientific Laboratory Supplies (SLS), containing the same target-element suite, was additionally used as an independent calibration-verification check. The use of calibration and verification standards from separate suppliers provided an additional check on the validity of the instrumental calibration.

Analytical performance deteriorated markedly at the lowest calibration levels. Back-calculation errors for the 0.114, 0.228 and 0.570 µg L^−1^ standards were +198.59%, +93.61% and +34.22%, respectively. The 1.141 µg L^−1^ calibrator showed an RSD of 4.00% and a back-calculation error of +15.64% and was therefore retained as the study-specific operational LLOQ. This value represents an operational analytical boundary for the reconstructed dataset and should not be interpreted as a formally validated mushroom-matrix-specific metrological limit of quantification. Complete calibration performance is provided in Table S2.

Calibration blanks, standards and samples contained ^115^In at a nominal concentration of 1 ng g^−1^ as a sequence-level internal-standard component of the analytical workflow. Within the Syngistix™ acquisition configuration, Ir (IS1) was assigned as the analyte-specific internal standard for ^192^Os. The documented ^115^In sequence response was therefore retained as an additional sequence-level instrumental diagnostic, whereas Ir (IS1) represents the analyte-specific internal-standard assignment used for Os measurement. The mushroom digestates were not subjected to additional automated prepFAST dilution after the manual ×11 dilution described above. No new Os-specific internal-standard acceptance criterion was imposed retrospectively.

Quality assurance comprised evaluation of calibration performance, instrumental washes, acid-digestion procedural blanks, internal-standard behaviour and an independently prepared aqueous platinum-group-element quality-control solution (QCC). The aqueous QCC had a nominal Os concentration of 231 µg L^−1^ and was analysed twice at the University of Oxford. Apparent Os concentrations were 249.524 and 253.743 µg L^−1^, giving a mean of 251.634 µg L^−1^, equivalent to 108.93% of the assigned concentration, with an RSD of 1.19% (*n* = 2).

Because the QCC concentration exceeded the highest Os calibrator used for the environmental samples (126.948 µg L^−1^), it was interpreted solely as a high-level instrumental response and repeatability check under extrapolated conditions. Furthermore, the aqueous QCC did not undergo the mushroom digestion and sample-preparation procedure. It therefore cannot establish within-range analytical trueness, digestion recovery, fungal-matrix recovery or overall Os mass balance.

Osmium-specific behaviour during sample preparation required additional consideration because Os can form volatile species under acidic and oxidising conditions. Dedicated ICP–MS methods have consequently employed post-digestion complexation or stabilisation approaches to minimise volatilisation-related analyte losses and analytical artefacts [25,26]. The present mushroom workflow employed closed-vessel, non-microwave HNO_3_/H_2_O_2_ digestion at 96 °C for 12 h, which may have reduced physical escape during the heated digestion stage relative to an open-vessel procedure. However, no Os-specific stabilisation step was incorporated into the analytical workflow. After cooling, the vessels were opened, and the digestates were filtered, diluted and subsequently prepared for ICP–MS analysis as described above. No Os-specific matrix-spike recovery experiment or independent mass-balance assessment was performed. Recovery of Os through digestion and subsequent sample handling was therefore not determined, and no retrospective recovery correction was applied.

Reference materials were incorporated within the wider Leicester multielement analytical programme. NIST SRM 1570a Trace Elements in Spinach Leaves (National Institute of Standards and Technology, Gaithersburg, MD, USA) and ERM-DB001 human hair (European Commission, Joint Research Centre, Institute for Reference Materials and Measurements, Geel, Belgium) were used to support quality assurance of the biological-matrix analytical workflow, while CRM059-50G Trace Metals—Loamy Clay 2 (Sigma-Aldrich RTC, Inc., Laramie, WY, USA) was used within the associated soil programme [23,35,36,37]. These materials provided matrix-based analytical quality assurance for elements carrying assigned concentrations. Although none of the available reference materials carried an assigned Os concentration, previously published validation of the same analytical programme demonstrated satisfactory recovery for other assigned elements; for example, recoveries for Cd, Cu and Zn were 89.7%, 102.4% and 97.5%, respectively, in NIST SRM 1570a, and 105.2%, 106.3% and 104.6%, respectively, in CRM059-50G [23,35]. Consequently, these materials supported the wider multielement analytical workflow but could not provide an Os-specific recovery estimate.

Accordingly, interpretation of Os was based on the documented calibration performance, internal-standard behaviour, procedural blanks, instrumental washes, aqueous QCC and analytical-background sensitivity framework described above.

### 4.6. Procedural Background and Operational Detection Criterion

Eighteen archived acid-digestion procedural-blank records were available within the analytical programme. The complete blank sequence was retained for analytical reconstruction and sensitivity assessment. The primary background definition was based on the procedure-defined post-replacement blank population, while alternative use of the complete archived blank population was evaluated separately as a sensitivity scenario (Section 4.8). The complete acid-digestion blank sequence and the definition of the primary background population are shown in Figure S1, with the corresponding numerical analytical-background data provided in Table S2.

Under the primary analytical interpretation (Scenario A), the procedural-background population comprised eight post-replacement acid-digestion blanks corresponding to tube IDs 121, 122, 140, 141, 159, 160, 178 and 179. The median ^192^Os response of these eight blanks was 0.2360029 µg L^−1^ and was retained as the Scenario A procedural-background reference value. The valid-blank variability used in construction of the operational detection criterion was 0.0010023 µg L^−1^.

Four instrumental wash measurements containing 2% HNO_3_ were retained as an independent measure of instrumental-background variability. These measurements produced an SD of 0.0017212 µg L^−1^.

Following adjustment for the applicable procedural-background reference value, the operational detection threshold on the introduced-solution scale was defined as the greater of three times the instrumental-wash variability and three times the valid-procedural-blank variability:

*T* = max(3 × SD_wash_, 3 × variability_blank_)

This yielded an operational detection threshold of: *T* = 0.0051637 µg L^−1^. Because 3 × SD_wash_ exceeded 3 × variability_blank_, the final operational detection threshold was wash-driven.

The study-specific solution-scale operational LLOQ of 1.141 µg L^−1^, established independently from calibration performance as described in Section 4.5, was retained as the quantitative analytical boundary. The operational detection threshold and operational LLOQ therefore represented distinct criteria: an analytical response could be distinguishable from the applicable operational background while remaining below the quantitative calibration boundary.

The common solution-scale operational detection threshold and operational LLOQ were converted to analytical-portion-specific dry-weight limits using the exact dry mass of each analytical portion, the 25 mL final digest volume and the ×11 manual pre-ICP–MS dilution factor, applied once.

Among the 153 included analytical portions, the resulting dry-weight operational detection thresholds ranged from 0.002795 to 0.177502 mg kg^−1^ dw, while the corresponding operational LLOQs ranged from 0.617667 to 39.221875 mg kg^−1^ dw.

These thresholds were treated as study-specific operational analytical criteria derived from the documented analytical workflow and were not interpreted as independently validated mushroom-matrix-specific metrological limits of detection or quantification.

### 4.7. Fruiting-Body Reconstruction and Analytical Classification

The 157 original analytical records included whole-fruiting-body measurements and separate cap and stem portions. Four unmatched tissue-only records could not be reconstructed to a complete fruiting body and were excluded.

The remaining 153 analytical portions comprised 89 whole-fruiting-body records and 64 portions forming 32 matched cap–stem pairs. Reconstruction produced 121 fruiting-body-level biological units.

At the analytical-portion level, background-adjusted responses at or below the applicable operational detection threshold were classified as ND. Responses above the threshold but below the 1.141 µg L^−1^ operational LLOQ were classified as detected below the operational LLOQ. A response satisfying the predefined quantitative calibration criterion would have been classified as quantified; no Os analytical portion ultimately supported a quantified fruiting-body classification.

Whole-fruiting-body analytical portions were retained directly as biological units. For matched cap–stem fruiting bodies, analytical information from both components was combined using the recorded dry masses of the analytical portions.

The component masses represent the quantities introduced into digestion and were used for analytical reconstruction only. They should not be interpreted as experimentally measured whole-fruiting-body cap:stem biomass ratios.

### 4.8. Analytical-Background Sensitivity Scenarios

Four predefined analytical-background scenarios were applied to evaluate the sensitivity of fruiting-body-level Os classification to alternative definitions of procedural or instrumental background. All scenarios were applied to the same reconstructed *N* = 121 fruiting-body-level biological units, and the study-specific operational LLOQ of 1.141 µg L^−1^ was retained unchanged throughout. The scenarios therefore differed in their treatment of analytical background rather than in the quantitative calibration criterion.

Scenario A constituted the primary analytical interpretation. It used the eight procedure-defined post-replacement acid-digestion blanks identified in Section 4.6 as the applicable procedural-background population.

Scenario B imposed a more restrictive batch-matched procedural-background criterion. A fruiting-body unit was evaluable under this scenario only when an eligible post-replacement acid-digestion procedural blank was available from the same analytical batch. Units for which this requirement could not be satisfied were classified as not evaluable rather than assigned an alternative background estimate.

Scenario C incorporated all 18 archived acid-digestion procedural blanks, including the earlier procedural phase characterised by elevated Os background. This scenario was retained as a deliberately conservative sensitivity boundary and was not treated as an alternative primary estimate of procedural background.

Scenario D used the retained instrumental wash measurements as an instrumental-background diagnostic comparator. Because these 2% HNO_3_ washes did not undergo mushroom digestion or sample preparation, they were not treated as procedural blanks and Scenario D was not interpreted as an alternative procedural-background model.

Analytical batches were retained using the coded identifiers B01–B10. Historical raw digestion-date labels were not used retrospectively to infer chronology, procedural status or analytical-background eligibility.

Classification agreement between Scenarios A and B was assessed only among fruiting-body units directly evaluable under both scenarios. Units not meeting the Scenario B batch-matching requirement were excluded from concordance calculations rather than treated as agreement or disagreement.

Scenario-specific outputs were subsequently compared at fruiting-body level using the same classification categories: ND, detected below the operational LLOQ, quantified, or not evaluable where applicable.

### 4.9. Statistical and Spatial Analysis

Analytical occurrence was summarised at the reconstructed fruiting-body level (*N* = 121) using counts and proportions. Exact two-sided 95% confidence intervals for binomial proportions were calculated using the Clopper–Pearson method [38].

Because no fruiting-body observation yielded a quantitatively valid Os result at or above the study-specific operational LLOQ, there were no uncensored quantitative observations from which a continuous Os concentration distribution could be estimated. Accordingly, no arithmetic or geometric means, medians, percentiles, regression-on-order-statistics estimates, Kaplan–Meier concentration estimates or parametric concentration-distribution models were generated. No replacement of non-detects or detected-below-operational-LLOQ observations by zero, fractions of an analytical limit, midpoint values or other numerical surrogates was performed [24].

Scenario A occurrence profiles were summarised descriptively by collection location, final taxonomic assignment, area type, geographical quadrant and analytical batch. Formal inferential comparisons among these groups were not undertaken because collection location, fungal taxon and analytical batch were strongly unbalanced and partly confounded. These outputs were therefore interpreted as descriptive and hypothesis-generating rather than as evidence of independent spatial or taxonomic effects.

Agreement between Scenarios A and B was evaluated only among the 59 fruiting-body units directly evaluable under both analytical-background definitions. Classification concordance was expressed as the proportion of identical classifications, with an exact binomial 95% confidence interval calculated using the Clopper–Pearson method. The 62 units not evaluable under Scenario B were excluded from the concordance denominator and were not treated as either concordant or discordant observations.

Spatial analysis incorporated all 121 audited fruiting-body units. Original point coordinates were retained in the World Geodetic System 1984 (WGS84) coordinate reference system and transformed for final static mapping to the British National Grid (EPSG:27700). Maps represented individual sampling-point analytical status only. No spatial interpolation, kriging, rasterised contamination surface or spatial concentration model was generated. Because numerically resolved site-level soil Os concentrations were unavailable and the topsoil composites were not spatially paired with individual fruiting bodies, no Os soil–mushroom correlation, concentration ratio, bioconcentration factor (BCF) or transfer factor was calculated.

All statistical analyses, analytical-data reconstruction, sensitivity analyses and reproducibility checks were performed in R version 4.4.2 (R Foundation for Statistical Computing, Vienna, Austria) [39]. The principal analytical workflow used the dplyr, tidyr, readr, ggplot2, scales, sf and patchwork packages.

The final analytical workflow was re-executed from the curated analytical dataset and reproduced, without numerical discrepancies, the complete reconstruction from 157 original analytical records to 153 included analytical portions and 121 fruiting-body-level biological units, together with the Scenario A–D analytical classifications, descriptive occurrence profiles and Scenario A–B concordance results. Package versions and complete R session information were retained as part of the reproducibility record.

### 4.10. Contextual Topsoil Programme

The mushroom survey formed part of a wider environmental-monitoring programme conducted across Leicester and Leicestershire that also included surface topsoils [23]. The soil component is included here solely to provide environmental context for the mushroom survey and was analysed independently from the fruiting-body-level Os occurrence framework described above.

Surface soil sampling targeted the upper 0–5 cm horizon. Across the wider programme, a total of 850 individual field increments were collected and pooled within sampling locations to generate 26 site-level composite topsoils. Each composite was homogenised, and two separately identified physical subsamples were subsequently retained for laboratory analysis, yielding 52 laboratory analytical records. These paired subsamples represented laboratory-level replication of the 26 site composites and were therefore not treated as 52 independent environmental samples; the environmental sample size remained *N* = 26 [23].

Quality assurance for the soil analytical programme included CRM059-50G Trace Metals—Loamy Clay 2. This material supported matrix-based quality assurance for elements carrying assigned concentrations within the wider soil programme. However, its certificate does not contain an assigned Os concentration, and it therefore could not be used to derive an Os-specific soil recovery estimate.

The site-level composite topsoils represented park-scale geochemical conditions rather than microscale substrates collected directly beneath individual mushroom fruiting bodies. In addition, numerically resolved site-level Os concentrations were not available in the final contextual topsoil dataset used for the present reconstruction.

Consequently, the topsoil dataset was retained exclusively as contextual information. It was not used to calculate Os soil–mushroom correlations, concentration ratios, bioconcentration factors (BCFs), bioaccumulation factors or other soil-to-fungus transfer metrics, and no causal inference regarding substrate-to-fruiting-body Os transfer was attempted.

### 4.11. Generative AI Assistance in Figure Preparation

OpenAI ChatGPT (version GPT-5.6 Sol), including its integrated image-generation functionality, was used during manuscript preparation to assist with the graphical design of the graphical abstract and Figure 3. The scientific content, wording, labels, interpretive boundaries and final composition of these graphical materials were defined and critically reviewed by the authors. No generative AI tool was used to generate, modify or analyse the underlying experimental data, analytical classifications, statistical outputs or numerical results.

## 5. Conclusions

Os-associated analytical responses were identified in a subset of wild mushroom fruiting bodies from Leicester and Leicestershire. Under the primary procedural-background definition, 28/121 fruiting-body units (23.1%; exact 95% CI: 16.0–31.7%) were classified as detected below the operational LLOQ, whereas 93/121 were non-detects and none yielded a quantitatively valid Os result. Classification was highly concordant for the 59 units directly comparable under the stricter same-batch background scenario (58/59), while the wash-derived and all-blank sensitivity scenarios demonstrated that occurrence classification at these ultratrace levels remains dependent on analytical-background definition. The principal environmental finding is therefore evidence of background-sensitive Os-associated occurrence rather than a quantitative concentration distribution.

Location- and taxon-specific occurrence profiles remain descriptive because of the unbalanced site–taxon–batch structure, and the available data do not support quantitative soil-to-fungus transfer, exposure or chemical-form-specific toxicological inference. Future targeted mushroom studies should combine substantially lower validated quantitative limits with Os-specific recovery control, high-sensitivity isotope-based determination, balanced sampling, paired substrate measurements and, where molecular or toxicological interpretation is intended, species-preserving chemical-speciation methods.

## Figures and Tables

**Table 1:** Analytical performance, background definition and quality-assurance parameters for ^192^Os determination in wild mushroom samples.

Parameter	Result/Interpretation
Monitored isotope	^192^Os
Instrumental platform	PerkinElmer NexION^®^ 2000B ICP–MS; Syngistix™ Software for ICP-MS version 3.2
Instrumental acquisition method	Original multielement Syngistix™ acquisition method used for mushroom digestates at the University of Oxford
^192^Os acquisition conditions	Peak hopping; standard mode; no collision/reaction gas; nominal mass 191.962; 20 sweeps/reading; 1 reading/replicate; 5 replicates; dwell time 25 ms per AMU; integration time 500 ms
Isobaric interference correction	Syngistix correction term −0.023153 × ^195^Pt applied to the ^192^Os response before data export
Calibration model	Linear regression of net intensity against nominal concentration, free intercept
Calibration points/range	10 non-zero standards; 0.114–126.948 µg L^−1^
Calibration coefficient of determination	*R*^2^ = 0.999925
Calibration linearity across the analytical programme	Laboratory-reported *R*^2^ values > 0.995 for all included elements
Independent calibration verification	Commercial multielement standard from Scientific Laboratory Supplies (SLS), independent of the primary QMx calibration standards and containing the same target-element suite
Study-specific operational LLOQ	1.141 µg L^−1^
Performance at defining LLOQ calibrator	RSD = 4.00%; back-calculation error = +15.64%
Interpretation of LLOQ	Study-specific operational LLOQ; not a formally validated matrix-specific metrological LoQ
Final digest volume/manual dilution	25 mL; ×11 manual pre-ICP–MS dilution applied once
Additional prepFAST dilution	None
Sequence-level internal-standard component	^115^In at a nominal concentration of 1 ng g^−1^ in calibration blanks, standards and samples; retained as a sequence-level instrumental diagnostic
Internal standard assigned to ^192^Os	Ir (IS1), as specified in the original Syngistix™ acquisition method
Primary procedural-background population	Eight post-replacement acid-digestion blanks: tube IDs 121, 122, 140, 141, 159, 160, 178 and 179
Scenario A procedural-background median	0.236003 µg L^−1^
Valid-blank variability used for threshold derivation	0.001002 µg L^−1^
Instrumental wash population/SD	*n* = 4; SD = 0.001721 µg L^−1^
Operational detection threshold	0.005164 µg L^−1^, defined as max(3 × wash SD, 3 × valid-blank variability); wash-driven
Dry-weight operational detection-threshold range	0.002795–0.177502 mg kg^−1^ dw
Dry-weight operational LLOQ range	0.617667–39.221875 mg kg^−1^ dw
Aqueous Os QCC result	249.524 and 253.743 µg L^−1^; mean 251.634 µg L^−1^ = 108.93% of assigned concentration; RSD = 1.19%; *n* = 2
Interpretation of aqueous QCC	High-level instrumental response/repeatability check under extrapolated conditions; not evidence of digestion recovery, matrix recovery or within-range analytical trueness
Digestion configuration	Closed-vessel, non-microwave acid digestion using HNO_3_/H_2_O_2_
Reference materials used in the wider analytical programme	NIST SRM 1570a Trace Elements in Spinach Leaves and ERM-DB001 human hair for the wider mushroom multielement programme; CRM059-50G Trace Metals—Loamy Clay 2 for the associated soil programme

The aqueous QCC was analysed above the calibration interval used for the environmental samples and did not undergo the mushroom digestion procedure; it was therefore used solely as a high-level instrumental performance and repeatability check. Reference materials supported quality assurance of the wider multielement analytical programme for elements carrying assigned values. Os-specific analytical interpretation and the limitations of the available quality-control materials are described in Section 4.5.

**Table 2:** Osmium analytical classification profile among 121 reconstructed fruiting-body units under Scenario A.

Classification	*n*	*N*	%	Exact 95% CI (%)
Non-detect	93	121	76.9	68.3–84.0
Detected below operational LLOQ	28	121	23.1	16.0–31.7
Quantified	0	121	0.0	0.0–3.0

**Table 3:** Sensitivity of fruiting-body-level Os classification to alternative analytical-background definitions.

Scenario	ND	Detected < Operational LLOQ	Not Evaluable	Quantified	Interpretation
A	93	28	0	0	Primary post-replacement procedural-background definition
B	56	3	62	0	Same-batch post-replacement procedural sensitivity
C	121	0	0	0	Conservative all-blank sensitivity
D	103	18	0	0	Wash-derived instrumental-background diagnostic

Among the 59 fruiting-body units directly comparable under Scenarios A and B, 58/59 classifications were concordant (98.3%; exact 95% CI: 90.9–100.0%). FB088 was the only discordant unit, changing from detected below the operational LLOQ under Scenario A to ND under Scenario B. Twenty-four of the 28 Scenario A detections were not evaluable under Scenario B.

**Table 4:** Published mushroom Os observations relevant to interpretation of the present study.

Study	Matrix/Context	Reported Os Information	Main Comparability Consideration
Rodushkin et al. [5]	Wild mushrooms, northeast Sweden	Mean values approximately 2.5 and 11 pg g^−1^; maximum values approximately 30 pg g^−1^ in Boletus edulis	Os-specific analytical approach incorporating separation as volatile OsO_4_ and gas-phase introduction into double-focusing sector-field ICP–MS, providing substantially greater pg g^−1^ sensitivity than the present multielement quadrupole ICP–MS method. Environmental and source-related differences also preclude direct concentration comparison.
Mleczek et al. [11]	20 wild mushroom species near a trafficked road, Poland	Approximately 0.01–0.02 mg kg^−1^ dw Os	Microwave-assisted HNO_3_ digestion followed by ICP-OES. Reported Os concentrations of approximately 0.01–0.02 mg kg^−1^ dw overlap part of the present sample-specific operational detection-threshold range but remain well below the present operational LLOQ range. This numerical overlap indicates potential detectability at concentrations of that order, not cross-method quantitative concordance.
Present study	Wild mushrooms from Leicester/Leicestershire green spaces	28/121 detected below operational LLOQ; 0/121 quantified	Background-aware occurrence classification; no continuous Os concentration distribution estimated.

## Data Availability

The data presented in this study are available in the Open Science Framework (OSF) repository, *Leicester topsoil and wild mushroom metal dataset*, at https://osf.io/xbrd7/overview?view_only=750aba9f380044e38ce5c9162e0d4020 (accessed on 15 August 2026). The repository includes curated CSV files containing raw and final calculated concentrations, limits of detection (LoDs) and below-LoD flags for the topsoil and wild mushroom datasets. The dataset is released under the Creative Commons Attribution 4.0 International licence (CC BY 4.0).

## Supplementary Materials

The following supporting information can be downloaded at https://www.mdpi.com/article/10.3390/ijms27188188/s1.
