# Supplementary material for: Ultratrace Osmium in Wild Mushrooms: Environmental Occurrence and Chemical-Form-Dependent Molecular and Toxicological Interpretation
Source: Int J Mol Sci. 2026 Sep 15;27(18):8188. doi: 10.3390/ijms27188188 (PMC13607779; doi:10.3390/ijms27188188)
Supplement: Supplementary file 1 [file ijms-27-08188-s001.zip › Supplementary Figures Os.pdf]

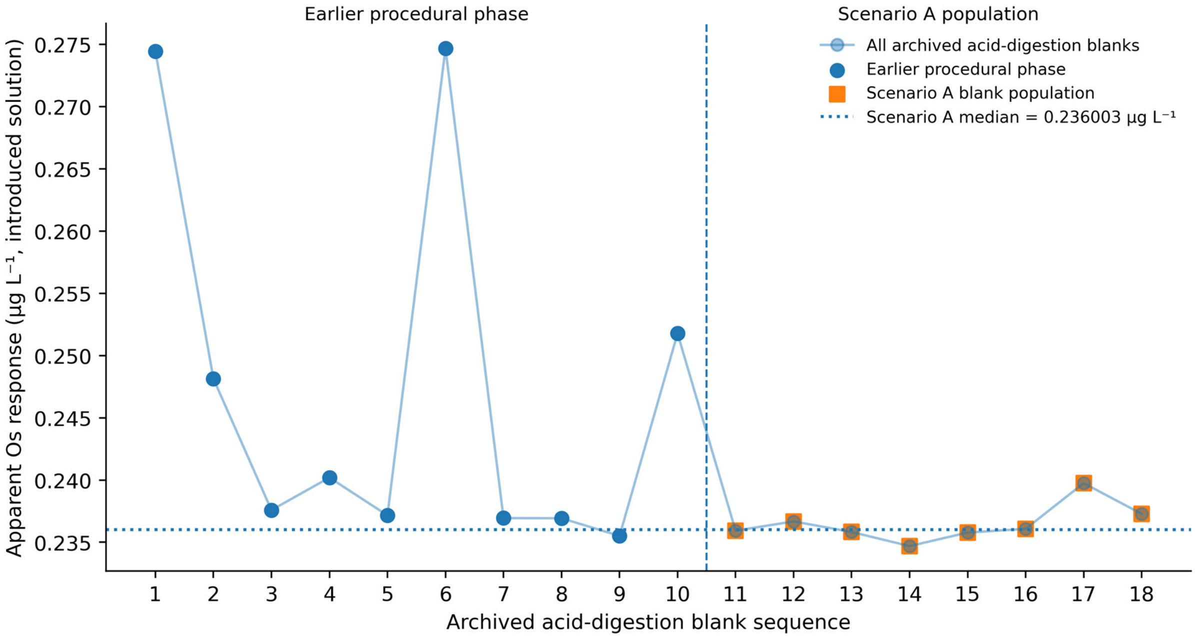

Figure S1. Archived acid-digestion blank sequence and primary Scenario A background definition. The first ten records belong to the documented earlier blank-preparation phase, whereas the final eight procedure-defined post-replacement acid-digestion blanks constitute the Scenario A background population (tube IDs 121, 122, 140, 141, 159, 160, 178 and 179). The horizontal dotted line indicates the Scenario A median response (0.236003 μg L^{-1}). Phase assignment follows the preserved procedural reconstruction; the exact date of vessel replacement was not documented contemporaneously. The figure shows why inclusion of the earlier blank phase materially changes ultratrace Os classification.

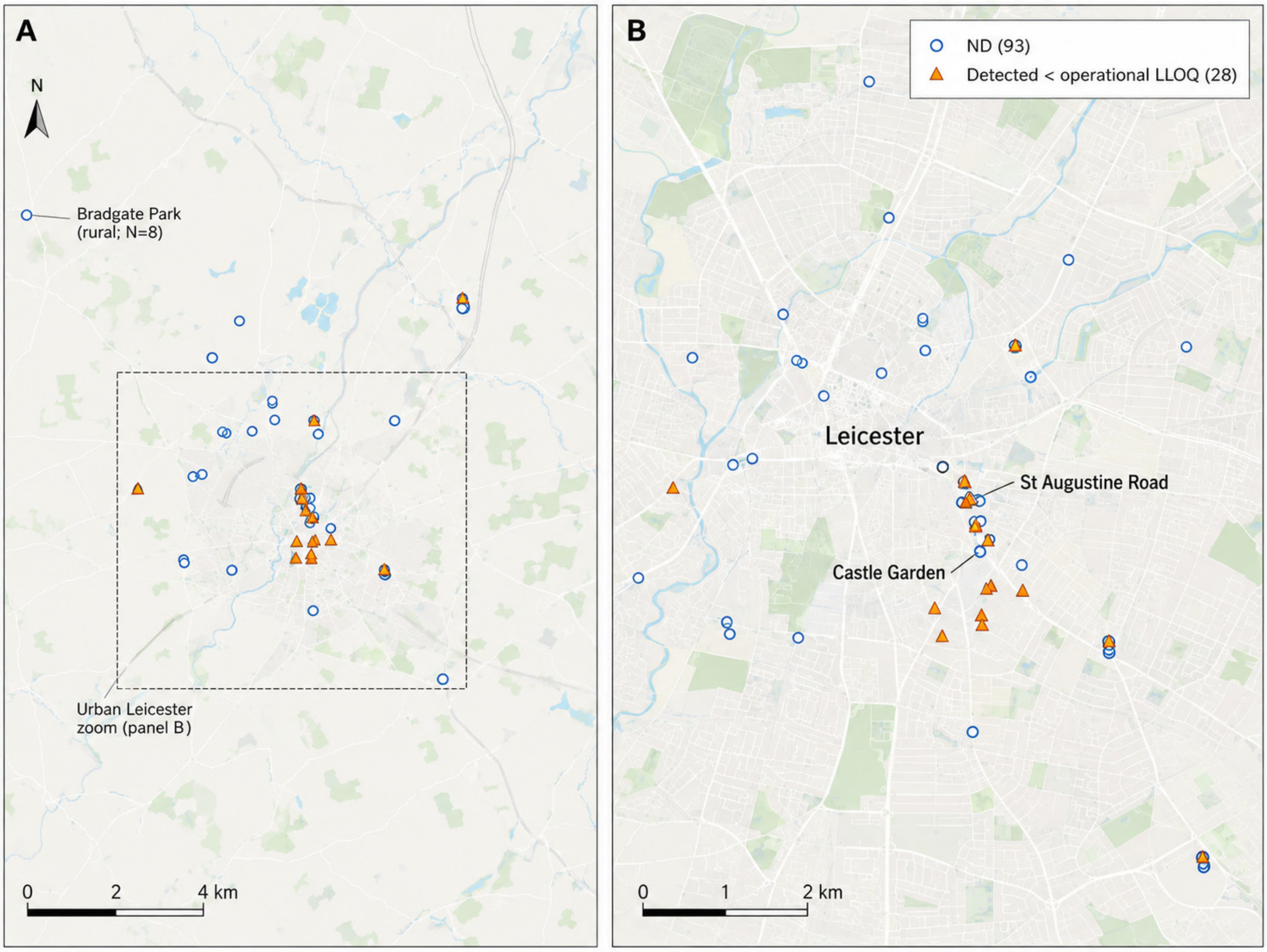

Figure S2. Spatial distribution of Os analytical status in wild mushroom fruiting bodies from Leicester and Leicestershire. (A) Full study extent, including the rural Bradgate Park observations; the dashed rectangle indicates the extent shown in panel B. (B) Enlarged view of the urban Leicester sampling area. All 121 reconstructed fruiting-body units are represented as either non-detect or detected below the operational LLOQ. Points are projected in British National Grid (EPSG:27700). No interpolation, kriging, contamination surface or soil-to-fungus transfer inference is applied.

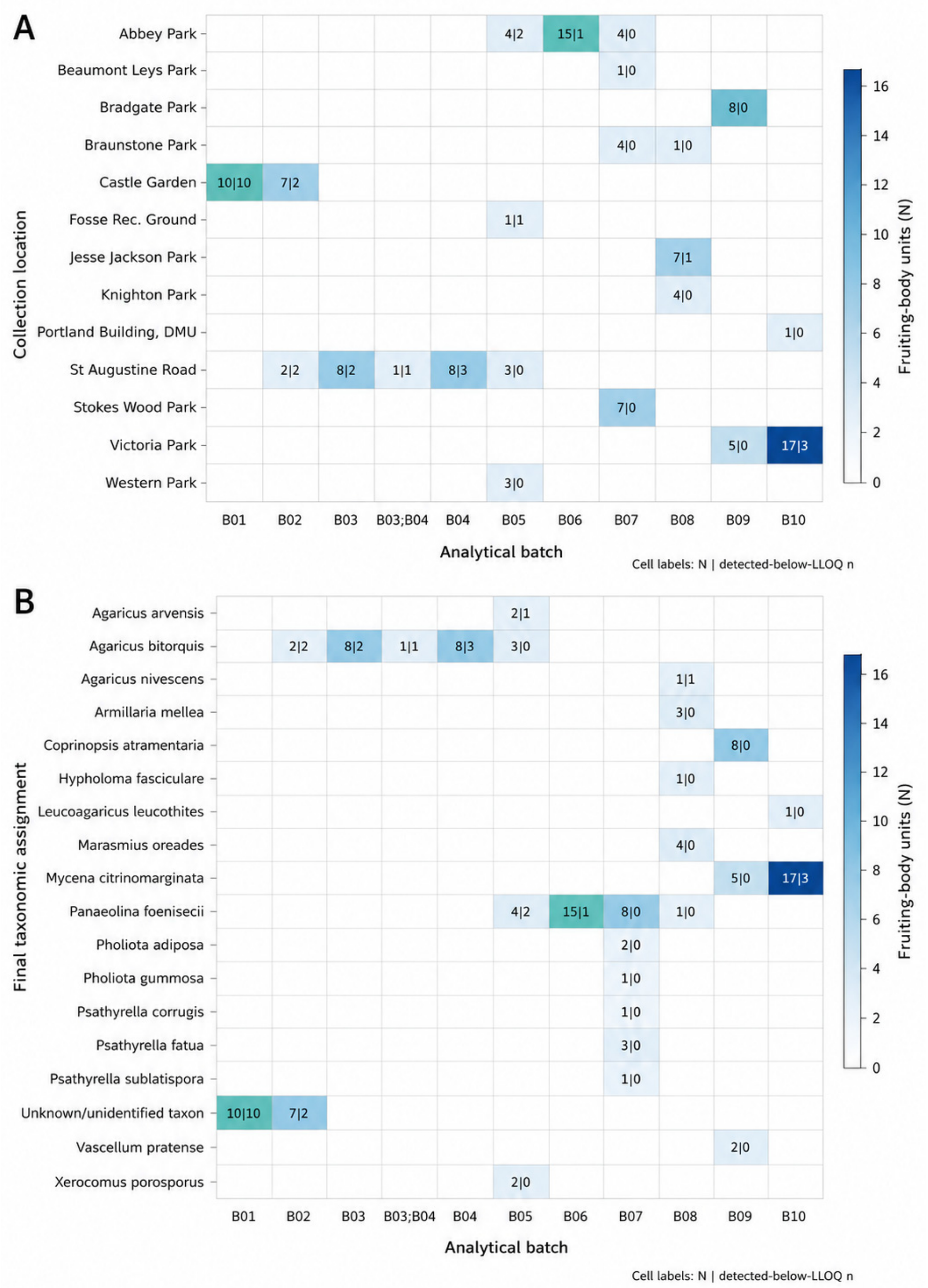

Figure S3. Analytical-design structure relevant to interpretation of location- and taxon-specific Os signal frequencies. (A) Collection location × analytical batch. (B) Final taxonomic assignment × analytical batch. Cell labels are N ∣ detected-below-operational-LLOQ n. The concentration of particular locations and taxa within restricted analytical batches demonstrates the unbalanced and partly confounded design; these matrices support descriptive, hypothesis-generating interpretation rather than independent site- or taxon-specific accumulation inference.
