# Supplementary material for: Ultratrace Osmium in Wild Mushrooms: Environmental Occurrence and Chemical-Form-Dependent Molecular and Toxicological Interpretation
Source: Int J Mol Sci. 2026 Sep 15;27(18):8188. doi: 10.3390/ijms27188188 (PMC13607779; doi:10.3390/ijms27188188)
Supplement: Supplementary file 1 [file ijms-27-08188-s001.zip › Supplementary Tables Os.pdf]

Table S1. Reconstruction of the original analytical dataset to the fruiting-body biological unit used for environmental interpretation.

Reconstruction step	*N*
Original analytical records	157
Excluded unmatched tissue-only records	4
Included analytical portions	153
Whole-fruiting-body measurements	89
Complete cap-stem pairs	32
Final fruiting-body biological units	121

Cap and stem analytical portions from the same fruiting body were not treated as independent mushrooms.

Table S2. Detailed ^{192}Os calibration, procedural-blank and instrumental-wash QA/QC supporting the operational analytical definitions.

Table S2A. Calibration performance.

Standard	Nominal (μg L^{-1})	Apparent (μg L^{-1})	Back-calculation error (%)	Operational LLOQ calibrator
Cal1	0.114	0.341	+198.59	No
Cal2	0.228	0.442	+93.61	No
Cal3	0.570	0.766	+34.22	No
Cal4	1.141	1.319	+15.64	Yes
Cal5	2.852	3.045	+6.79	No
Cal6	5.078	5.070	-0.16	No
Cal7	10.156	9.958	-1.95	No
Cal8	25.390	24.544	-3.33	No
Cal9	50.779	50.557	-0.44	No
Cal10	126.948	127.215	+0.21	No

Table S2B. Archived acid-digestion blank sequence.

Sequence	Tube ID	Batch	Procedural phase	Apparent Os (μg L^{-1})	Eligible for Scenario A/B
1	14	B01	Earlier phase	0.274449	No
2	30	B02	Earlier phase	0.248142	No
3	45	B03	Earlier phase	0.237584	No
4	46	B03	Earlier phase	0.240195	No
5	64	B04	Earlier phase	0.237163	No
6	65	B04	Earlier phase	0.274680	No
7	83	B05	Earlier phase	0.236941	No
8	84	B05	Earlier phase	0.236928	No
9	102	B06	Earlier phase	0.235512	No
10	103	B06	Earlier phase	0.251777	No
11	121	B07	Scenario A population	0.235929	Yes
12	122	B07	Scenario A population	0.236675	Yes
13	140	B08	Scenario A population	0.235825	Yes
14	141	B08	Scenario A population	0.234692	Yes
15	159	B09	Scenario A population	0.235780	Yes
16	160	B09	Scenario A population	0.236077	Yes
17	178	B10	Scenario A population	0.239748	Yes
18	179	B10	Scenario A population	0.237277	Yes

Table S2C. Instrumental wash measurements.

Measurement	Introduced-solution response (μg L^{-1})	Control
1	0.237587	instrumental wash
2	0.237003	instrumental wash
3	0.240751	instrumental wash
4	0.239450	instrumental wash

QA/QC summary: calibration $R^2 = 0.999925$; study-specific operational LLOQ = 1.141 μg L^{-1} (defining calibrator RSD = 4.00%; back-calculation error = +15.64%). Scenario A procedural-background median = 0.236003 μg L^{-1}; valid-blank variability = 0.001002 μg L^{-1}; wash SD = 0.001721 μg L^{-1}. The operational detection threshold was 0.005164 μg L^{-1} = max(3 × wash SD, 3 × valid-blank variability), and was therefore wash-driven. The operational LLOQ is study-specific and is not a formally validated matrix-specific metrological LoQ.

Table S3. Complete descriptive Scenario A Os occurrence profiles with exact Clopper–Pearson 95% confidence intervals. All group-level summaries are descriptive only.

Table S3A. Collection-location profile.

Group	*N*	Detected < operational LLOQ (*n*)	ND (*n*)	Signal (%)	Exact 95% CI (%)
Abbey Park	23	3	20	13.0	2.8–33.6
St Augustine Road	22	8	14	36.4	17.2–59.3
Victoria Park	22	3	19	13.6	2.9–34.9
Castle Garden	17	12	5	70.6	44.0–89.7
Bradgate Park	8	0	8	0.0	0.0–36.9
Jesse Jackson Park	7	1	6	14.3	0.4–57.9
Stokes Wood Park	7	0	7	0.0	0.0–41.0
Braunstone Park	5	0	5	0.0	0.0–52.2
Knighton Park	4	0	4	0.0	0.0–60.2
Western Park	3	0	3	0.0	0.0–70.8
Beaumont Leys Park	1	0	1	0.0	0.0–97.5
Fosse Rec. Ground	1	1	0	100.0	2.5–100.0
Portland Building, DMU	1	0	1	0.0	0.0–97.5

Table S3B. Final taxonomic-assignment profile.

Group	*N*	Detected < operational LLOQ (*n*)	ND (*n*)	Signal (%)	Exact 95% CI (%)
Panaeolina foenisecii	28	3	25	10.7	2.3–28.2
Agaricus bitorquis	22	8	14	36.4	17.2–59.3
Mycena citrinomarginata	22	3	19	13.6	2.9–34.9
Unknown/unidentified taxon	17	12	5	70.6	44.0–89.7
Coprinopsis atramentaria	8	0	8	0.0	0.0–36.9
Marasmius oreades	4	0	4	0.0	0.0–60.2
Armillaria mellea	3	0	3	0.0	0.0–70.8
Psathyrella fatua	3	0	3	0.0	0.0–70.8
Agaricus arvensis	2	1	1	50.0	1.3–98.7
Pholiota adiposa	2	0	2	0.0	0.0–84.2
Vascellum pratense	2	0	2	0.0	0.0–84.2
Xerocomus porosporus	2	0	2	0.0	0.0–84.2
Agaricus nivescens	1	1	0	100.0	2.5–100.0
Hypholoma fasciculare	1	0	1	0.0	0.0–97.5
Leucoagaricus leucothites	1	0	1	0.0	0.0–97.5
Pholiota gummosa	1	0	1	0.0	0.0–97.5
Psathyrella corrugis	1	0	1	0.0	0.0–97.5
Psathyrella sublatispora	1	0	1	0.0	0.0–97.5

Table S3C. Area-type profile.

Group	*N*	Detected < operational LLOQ (*n*)	ND (*n*)	Signal (%)	Exact 95% CI (%)
Urban	113	28	85	24.8	17.1–33.8
Rural	8	0	8	0.0	0.0–36.9

Table S3D. Geographical-quadrant profile.

Group	*N*	Detected < operational LLOQ (*n*)	ND (n)	Signal (%)	Exact 95% CI (%)
NW	59	21	38	35.6	23.6–49.1
NE	30	4	26	13.3	3.8–30.7
SE	27	3	24	11.1	2.4–29.2
SW	5	0	5	0.0	0.0–52.2

Table S3E. Analytical-batch profile.

Group	*N*	Detected < operational LLOQ (*n*)	ND (*n*)	Signal (%)	Exact 95% CI (%)
B10	18	3	15	16.7	3.6–41.4

Group	*N*	Detected < operational LLOQ (*n*)	ND (*n*)	Signal (%)	Exact 95% CI (%)
B07	16	0	16	0.0	0.0–20.6
B06	15	1	14	6.7	0.2–31.9
B09	13	0	13	0.0	0.0–24.7
B08	12	1	11	8.3	0.2–38.5
B05	11	3	8	27.3	6.0–61.0
B01	10	10	0	100.0	69.2–100.0
B02	9	4	5	44.4	13.7–78.8
B03	8	2	6	25.0	3.2–65.1
B04	8	3	5	37.5	8.5–75.5
B03;B04	1	1	0	100.0	2.5–100.0

Interpretation: Park, taxon, area type, quadrant and analytical batch were unbalanced and partly confounded. These frequencies are retained as descriptive profiles and do not support causal enrichment or intrinsic accumulation claims.

Table S4. Scenario A–B concordance summary for the directly comparable fruiting-body subset.

**Scenario A**	**Scenario B**	***n***
ND	ND	55
ND	DETECTED_BELOW_LLOQ	0
DETECTED_BELOW_LLOQ	ND	1
DETECTED_BELOW_LLOQ	DETECTED_BELOW_LLOQ	3

Concordance metrics.

**Metric**	**Result**
Comparable A-B units	59
Concordant A-B units	58
Concordance percent	98.3% (exact 95% CI: 90.9–100.0%)
Discordant unit	FB088
FB088 transition	DETECTED_BELOW_LLOQ -> ND
Scenario B not evaluable	62

The A–B agreement applies only to the 59 directly comparable fruiting-body units and should not be interpreted as independent validation of all 28 Scenario A detections.

Table S5. Group-level analytical-confounding and inferential-eligibility assessment.

Dimension	Group	*N*	Batches (*n*)	Parks (*n*)	Taxa (*n*)	Eligibility
Park	Castle Garden	17	2	1	1	Descriptive Only
	Western Park	3	1	1	2	Descriptive Only
	Abbey Park	23	3	1	1	Descriptive Only
	Stokes Wood Park	7	1	1	4	Descriptive Only
	Beaumont Leys Park	1	1	1	1	Descriptive Only
	Braunstone Park	5	2	1	1	Descriptive Only
	Jesse Jackson Park	7	1	1	3	Descriptive Only
	Knighton Park	4	1	1	2	Descriptive Only
	Bradgate Park	8	1	1	1	Descriptive Only
	Victoria Park	22	2	1	1	Descriptive Only
	Portland Building, DMU	1	1	1	1	Descriptive Only
	Fosse Rec. Ground	1	1	1	1	Descriptive Only
	St Augustine Road	22	5	1	1	Descriptive Only
Taxon	Unknown/unidentified taxon	17	2	1	1	Descriptive Only
	Xerocomus porosporus	2	1	1	1	Descriptive Only
	Panaeolina foenisecii	28	4	2	1	Descriptive Only
	Pholiota adiposa	2	1	1	1	Descriptive Only
	Psathyrella sublatispora	1	1	1	1	Descriptive Only
	Pholiota gummosa	1	1	1	1	Descriptive Only
	Psathyrella fatua	3	1	1	1	Descriptive Only
	Psathyrella corrugis	1	1	1	1	Descriptive Only
	Vascellum pratense	2	1	1	1	Descriptive Only
	Marasmius oreades	4	1	1	1	Descriptive Only
	Hypholoma fasciculare	1	1	1	1	Descriptive Only
	Coprinopsis atramentaria	8	1	1	1	Descriptive Only
	Mycena citrinomarginata	22	2	1	1	Descriptive Only
	Leucoagaricus leucothites	1	1	1	1	Descriptive Only
	Agaricus arvensis	2	1	2	1	Descriptive Only
	Agaricus bitorquis	22	5	1	1	Descriptive Only
	Agaricus nivescens	1	1	1	1	Descriptive Only
	Armillaria mellea	3	1	1	1	Descriptive Only
Area type	Urban	113	11	12	17	Descriptive Only
	Rural	8	1	1	1	Descriptive Only
Quadrant	NW	59	8	7	10	Descriptive Only
	NE	30	4	2	4	Descriptive Only
	SW	5	2	1	1	Descriptive Only
	SE	27	3	3	4	Descriptive Only

All listed group comparisons were classified as descriptive only because location, taxonomic composition and analytical batch were not independently balanced. Exact park × batch and taxon × batch counts are shown in Figure S3.
